# Multimodal fusion analysis of structural connectivity and gray matter morphology in migraine

**DOI:** 10.1002/hbm.25267

**Published:** 2020-12-08

**Authors:** Álvaro Planchuelo‐Gómez, David García‐Azorín, Ángel L. Guerrero, Santiago Aja‐Fernández, Margarita Rodríguez, Rodrigo de Luis‐García

**Affiliations:** ^1^ Imaging Processing Laboratory Universidad de Valladolid Valladolid Spain; ^2^ Headache Unit, Department of Neurology Hospital Clínico Universitario de Valladolid Valladolid Spain; ^3^ Institute for Biomedical Research of Salamanca (IBSAL) Salamanca Spain; ^4^ Department of Medicine Universidad de Valladolid Valladolid Spain; ^5^ Department of Radiology Hospital Clínico Universitario de Valladolid Valladolid Spain

**Keywords:** brain, connectome, diffusion magnetic resonance imaging, magnetic resonance imaging, migraine disorders

## Abstract

No specific migraine biomarkers have been found in single‐modality MRI studies. We aimed at establishing biomarkers for episodic and chronic migraine using diverse MRI modalities. We employed canonical correlation analysis and joint independent component analysis to find structural connectivity abnormalities that are related to gray matter morphometric alterations. The number of streamlines (trajectories of estimated fiber‐tracts from tractography) was employed as structural connectivity measure, while cortical curvature, thickness, surface area, and volume were used as gray matter parameters. These parameters were compared between 56 chronic and 54 episodic migraine patients, and 50 healthy controls. Cortical curvature alterations were associated with abnormalities in the streamline count in episodic migraine patients compared to controls, with higher curvature values in the frontal and temporal poles being related to a higher streamline count. Lower streamline count was found in migraine compared to controls in connections between cortical regions within each of the four lobes. Higher streamline count was found in migraine in connections between subcortical regions, the insula, and the cingulate and orbitofrontal cortex, and between the insula and the temporal region. The connections between the caudate nucleus and the orbitofrontal cortex presented worse connectivity in chronic compared to episodic migraine. The hippocampus was involved in connections with higher and lower number of streamlines in chronic migraine. Strengthening of structural networks involving pain processing and subcortical regions coexists in migraine with weakening of cortical networks within each lobe. The multimodal analysis offers a new insight about the association between brain structure and connectivity.

## Graphical abstract

Planchuelo‐Gómez et al. associate structural connectivity with gray matter morphometry. In chronic and episodic migraine, strengthened connectivity with pain processing regions, and higher cortical curvature related to weakened connectivity within each lobe were found. These results give a new insight about migraine neuroimaging biomarkers.

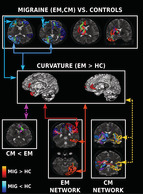

**Abbreviations**
BOLD: blood oxygen level dependent
CM: chronic migraine
DTI: diffusion tensor imaging
DWI: diffusion‐weighted image
EM: episodic migraine
FIT: fusion ICA toolbox
fMRI: functional MRI
GUI: guide user interface
HC: healthy controls
jICA: joint independent component analysis
mCCA: multimodal canonical correlation analysis
PCA: principal component analysis
TE: echo time
TFE: turbo field echo
TR: repetition time

## 1 INTRODUCTION

Migraine is a primary headache disorder characterized by recurrent attacks with unilateral, pulsatile and moderate or severe intensity, lasting from four to 72 hr (Headache Classification Committee of the International Headache Society, 2018). Migraine can be divided into two types: episodic migraine (EM) and chronic migraine (CM). CM is characterized by headache occurring on 15 or more days per month for more than 3 months during which, on at least eight of these days, the headache has migrainous characteristics (Headache Classification Committee of the International Headache Society, 2018), while EM patients suffer from headache during less than 15 days per month. The number of headache days per month is the unique criterion to distinguish between both types and, currently, there is no specific CM biomarker.

The migrainous brain has been studied in vivo, using different imaging techniques, in several neuroimaging studies. The vast majority of these studies are based on the separated analysis of individual MRI modalities. In the interictal phase of migraine, structural and functional changes in EM and CM have been observed in regions involved in pain such as the hippocampus and the cingulate cortex, with connectivity alterations which might influence multisensory integration, pain experience, and chronification (Messina, Filippi, & Goadsby, 2018). A meta‐analysis reported decreased gray matter volume in migraine compared to controls in several regions (Jia & Yu, 2017), although the results may be controversial (Burke et al., 2020). Functional and diffusion MRI have also been employed separately to characterize migraine, but no migraine biomarkers could be identified (Skorobogatykh et al., 2019) or the results between studies are conflicting. CM patients have shown higher functional connectivity in the pain matrix compared to EM (Lee et al., 2019), and alterations in structural connectivity involving regions like the putamen or the temporal cortex have been found (Planchuelo‐Gómez et al., 2020a). With respect to separate gray and white matter alterations, on the one hand, decreased axial diffusivity in diverse white matter tracts (Planchuelo‐Gómez et al., 2020b) and reduced cortical thickness, gray matter volume and area have been found in CM (Planchuelo‐Gómez, García‐Azorín, Guerrero, Rodríguez, et al., 2020). On the other hand, no significant differences between CM and EM have been found using diffusion tensor imaging (DTI) and voxel‐based morphometry independently (Neeb et al., 2015, 2017).

Although differences between patients and controls in diverse individual MRI modalities have been reported, it is essential to assess simultaneously data from different sources to understand migraine pathophysiology. Changes observed using different modalities are likely different aspects of a more complex brain alteration pattern that cannot be completely understood using a single modality. It is therefore of paramount importance to assess the existing relationships between these independently found alterations to create a more global picture of the pathophysiology of migraine.

Some authors have previously studied change patterns combining different imaging techniques. The simplest method is a direct correlation analysis between different modalities. In migraine with aura patients, resting state activity has been found to be correlated with fractional anisotropy and radial diffusivity in the corpus callosum (Faragó et al., 2019). However, note that the whole relationship is not totally resolved, since the correlation method does not analyze covariance patterns between the assessed modalities.

More sophisticated approaches have been used to assess other neurological disorders from this perspective. The most employed techniques are those based on canonical correlation analysis (CCA) and independent component analysis (ICA). Multimodal CCA (mCCA) followed by joint ICA (jICA) has allowed to perform a simultaneous analysis of images or maps from different modalities (Sui et al., 2011), including tissue types such as gray and white matter. It has been used to characterize patients with schizophrenia (Lottman et al., 2018; Sui et al., 2011, 2013), Alzheimer's disease (Ouyang et al., 2015) and obsessive–compulsive disorder (Kim, Jung, Kim, Jang, & Kwon, 2015). Furthermore, other techniques have been employed to assess healthy controls (HCs), such as hybrid connICA (Amico & Goñi, 2018) and linked ICA (Llera, Wolfers, Mulders, & Beckmann, 2019). Nevertheless, none of these methods has been used to analyze migraine.

The aim of this study is to investigate alteration patterns that may arise from the joint analysis of gray matter morphology and structural connectivity. From the identification of diverse morphological and structural connectivity patterns, our objective was the obtention of biomarkers for EM and CM compared to controls, and of specific CM biomarkers. To that end, mCCA‐jICA was employed. To the best of our knowledge, this is the first study to combine neuroimaging data from these two sources using this kind of approach, as well as the first study to focus on migraine using a modality fusion methodology. We hypothesize that the joint analysis of gray matter morphology and structural connectivity will allow the discovery of new alteration patterns that cannot be found using a single modality.

## 2 MATERIALS AND METHODS

### 2.1 Participants

We included 160 subjects, divided in 50 HC, 54 EM patients, and 56 CM patients. The same subjects were included in previous studies (Planchuelo‐Gómez, et al., 2020a, 2020b). The inclusion and exclusion criteria for patients and controls were the same that were described in both studies and can be found in Supplementary File 1.

The patients kept a headache diary in the 3 months prior to the MRI acquisition and were in stable situation when they were included in the study. This diary was used to evaluate headache and migraine frequency. The frequency values of the month of the scan were taken as reference for quantitative analysis. Patients with high frequency EM (10–14 headache days per month) were discarded to avoid confusion with EM or CM. More details can be found in Supplementary File 1.

To ensure that the controls suffered neither migraine nor headache with migrainous features, a questionnaire was provided to the HC. The questionnaire included questions related to previous diagnosis of migraine, made by a neurologist or a primary care physician, and migraine features according to the criteria C and D of Migraine without aura from the third edition of the International Classification of Headache Disorders (Headache Classification Committee of the International Headache Society, 2018). Together with questions related to the Criterion C, we also included a specific question related to the frequency of headache in more than 15 days per month. To evaluate the intensity of pain (Criterion C), the subject was asked whether an activity should be stopped, or needed to lay in bed, at least for 2 hr because of headache. In case of unclear situation with a control, a neurologist specialized in headache disorders was asked to clarify the inclusion of the subject in the study.

### 2.2 MRI acquisition

In the same session, high‐resolution 3D T1‐ and diffusion‐weighted images (DWI) were acquired using a Philips Achieva 3 T MRI unit (Philips Healthcare, Best, The Netherlands) with a 32‐channel head coil.

For the anatomical T1‐weighted images, the following acquisition parameters were used: Turbo Field Echo sequence, repetition time (TR) = 8.1 ms, echo time (TE) = 3.7 ms, flip angle = 8°, 256 × 256 matrix size, 1 × 1 × 1 mm^3^ of spatial resolution and 160 slices covering the whole brain.

DWI were acquired using the next parameters: TR = 9,000 ms, TE = 86 ms, flip angle = 90°, 61 gradient directions, one baseline volume, *b*‐value = 1,000 s/mm^2^, 128 × 128 matrix size, 2 × 2 × 2 mm^3^ of spatial resolution and 66 axial slices covering the whole brain.

The acquisition protocol was the same that was used in Planchuelo‐Gómez, et al. (2020a, 2020b). Further details can be found in Supplementary File 1.

### 2.3 Features estimation

Two groups of variables were used as features to describe gray matter structure and the connections between the gray matter regions. The first group was composed of four gray matter morphometric characteristics: cortical curvature, cortical thickness, surface area, and gray matter volume. The cortical curvature is related to the folding of the cortex, while the cortical thickness and the surface area are strongly related to the gray matter volume. On the other hand, the number of streamlines was used as a parameter to represent structural connectivity. The streamlines are the trajectories of white matter fiber tracts that are estimated with a tractography algorithm.

The analysis of this study was based on the comparison of these five measures between patients with EM and CM, and HC, after the application of a multimodal fusion procedure. The obtention of the features is briefly explained on the following two subsections.

#### 2.3.1 Morphometric gray matter features

From the T1‐weighted images, cortical curvature, cortical thickness and surface area were obtained for 68 cortical regions from the Desikan–Killiany atlas (Desikan et al., 2006), and gray matter volume was also calculated for the previous 68 regions plus 16 subcortical regions from the same atlas. The full image processing is described in (Planchuelo‐Gómez, García‐Azorín, Guerrero, Rodríguez, et al., 2020) and Supplementary File 1. The FreeSurfer (https://surfer.nmr.mgh.harvard.edu) automatic cortical parcellation pipeline was used to extract the gray matter features.

For each subject, the output of this analysis was composed of three 1×68 vectors, referring to curvature, thickness and area in the 68 cortical, and one 1×84 vector with the gray matter volume values in the 68 cortical plus 16 subcortical regions.

#### 2.3.2 Structural connectivity

The T1‐weighted images and the DWI data were employed to get structural connectivity matrices. The full processing pipeline, including DWI preprocessing, is described in (Planchuelo‐Gómez, et al., 2020a) and Supplementary File 1.

Briefly, using MRtrix tools (Tournier et al., 2019), anatomically‐constrained tractography (10 million streamlines per subject) was performed after estimating the fiber orientation distributions from the DWIs and the “five‐tissue‐type” segmented images from the T1‐weighted images (Smith, Tournier, Calamante, & Connelly, 2012).

As a result of this analysis, a symmetric 84×84 matrix was obtained for each subject. Connections with less than 1,000 streamlines (group mean) in the three groups of study were discarded. After the removal of these weak connections, the result was a 1×620 feature vector per subject.

### 2.4 mCCA and jICA

The main assumption of this method is that the multimodal dataset is a linear mixture of mixing profiles and independent sources. The fusion of mCCA and jICA allows to overcome the limitations of both methods when they are used independently. mCCA provides an initial estimation for jICA, and the components from each modality are linked due to the maximum correlation across the datasets assumed by the canonical variates from CCA (Correa, Li, Adali, & Calhoun, 2008).

The mCCA‐jICA method was first developed in (Sui et al., 2011) for the joint analysis of gray and white matter in schizophrenia. The Fusion ICA Toolbox (FIT, http://trendscenter.org/software/fit/), version 2.0d, was employed to implement the method. Using this tool, images, image‐like data (e.g., DTI maps) or other type or data such as EEG or genetic data can be used as an input. In our case, the feature vectors described before for gray matter morphology and structural connectivity were employed.

Before the description of the mCCA‐jICA method, it is important to explain two concepts: The principal components and the canonical variants.

The principal components are the result of a decomposition technique, the principal component analysis (PCA). The objective of this method is to summarize the information from a large dataset into a set of uncorrelated variables that at the same time keep a maximized variance. In other words, PCA aims at explaining the variability of a dataset using few variables. These variables are the principal components, which are ordered from highest to lowest explained variance.

ICA, which is the technique actually employed in this study, is somehow similar to PCA. However, in ICA the independent components that are obtained are not only uncorrelated as the principal components from PCA, but also do not hold any higher order dependence. Finally, in contrast to PCA, the independent components from ICA are equally important.

On the other hand, CCA is a method employed to identify and quantify relationships between two datasets using few variables. For each dataset, there is a group of canonical variants (the result of CCA) that explain the variability within a dataset and between the two datasets. Each pair of canonical variants is independent from other pairs, and the pairs are ordered from highest to lowest correlation.

The procedure we have employed is as follows:

1. *Determination of number of components*: Before starting mCCA, the number of principal components for each modality and the number of independent components should be elucidated. In the original method (Sui et al., 2011), the number of components was obtained using the minimum description length criteria (Li, Adali, & Calhoun, 2007). This method is suitable for images, with thousands or millions of elements (pixels or voxels), but our data dimensionality is relatively small (in the order of hundreds), so we decided to use alternative criteria.
  To determine the number of components from each modality, we followed the Horn's test (Horn, 1965). We computed the eigenvalues from the original data and randomly generated data of the same dimensionality. The eigenvalues from the random data were obtained for 500 generated datasets. Each eigenvalue from the original data was then compared to the corresponding eigenvalue from the random data, taking the 95th percentile value from the 500 random datasets as the eigenvalue to be compared. The number of components to retain in each feature was equal to the number of eigenvalues from the original dataset greater than the 95th percentile corresponding eigenvalue from the random data.
2. *Determination of number of canonical variants*: Once the number of components for each feature was obtained, the number of canonical variants is equal to the minimum number of components of the features that were analyzed.
3. *Dimension reduction on the data using singular value decomposition*: We follow the same procedure implemented in Sui et al. (2011), with the necessary adaptations to our data (more details in Supplementary File 2).
4. *mCCA followed by jICA*: Similar to Sui et al. (2011), described in detail in Supplementary File 2.

**FIGURE 1:**
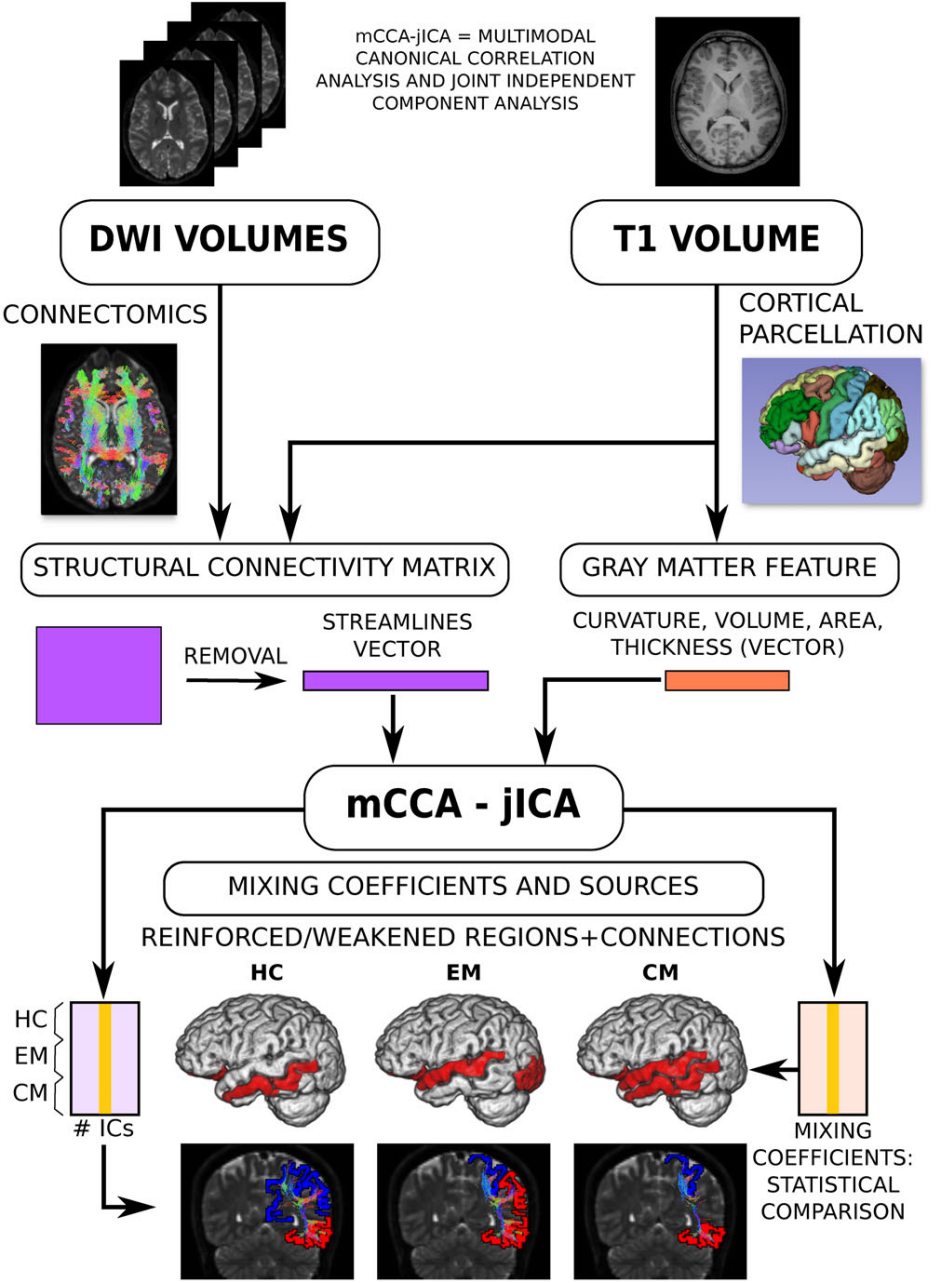
Overview of the method multimodal canonical correlation analysis‐joint independent component analysis (mCCA‐jICA). Schematic diagram showing the steps from the use of features from MRI volumes to the obtention of mixing coefficients and spatial sources representing the jICA components from structural connectivity and gray matter morphometry. Regions and connections shown here have only illustrative purpose and should not be interpreted as results

A summary of the whole procedure is shown in Figure 1.

The results from this procedure were:

- A matrix of mixing coefficients, with values for each subject and component. This result determines the weight of a modality in each group.
- The source components represented as Z‐scores for each study group (HC, EM, and CM) and modality (gray matter morphology or structural connectivity), with one value per region or connection. This result represents the regions or connections which are more strengthened or weakened in each group. The components were sorted from highest to lowest correlation between the mixing coefficients from each modality, that is, IC1 has the highest correlation between the mixing coefficients from each modality, and the last IC has the lowest correlation.

### 2.5 Statistical analysis

There are obvious relations between the different gray matter morphology features. For instance, the gray matter volume of a cortical region is correlated to its thickness and area. Because of this, considering all gray matter morphology features together in the mCCA‐jICA pipeline would yield mixing coefficients that are not independent.

Because of this, a separate mCCA‐jICA full procedure was performed for each gray matter morphometric feature, in order to assess the relationship of structural connectivity with each feature. Therefore, there were four main sets of results:

1. Curvature and connectivity.
2. Thickness and connectivity.
3. Surface area and connectivity.
4. Gray matter volume and connectivity.

Due to the absence of subcortical values of curvature, thickness, and area, in these situations we discarded the connections where only subcortical regions were involved. Hence, the input corresponding to the structural connectivity was a 1×570 vector instead of a 1×620 vector in these cases.

As secondary analysis, we repeated the same procedure with all the gray matter morphometric parameters, except gray matter volume, to assess possible relationships between them (because of evident reasons, volume is related to thickness and area).

#### 2.5.1 Mixing coefficients

The ICA loadings or mixing coefficients quantify the weight of the pattern represented by the independent component of each modality in each subject. Comparing these coefficients between the groups, it can be checked whether a specific pattern from an independent source or component is reinforced in patients with respect to controls and vice versa.

If mixing coefficients from a specific component were significantly different between groups in both modalities, this would be a joint component. This kind of components shows a joint variance across modalities (Stephen et al., 2013) and changes between groups in one modality would be linked to changes in another modality.

The alternative situation occurs when significant differences between groups are found only in one modality. In this case, the corresponding components would be modal‐specific. These components can differentiate the groups using the data from one modality (Lottman et al., 2018) and modifications from the modality where significant differences were found would not be linked a priori to changes in other assessed modalities.

Normality and homogeneity of variance of the mixing coefficients were analyzed with the Kolmogorov–Smirnov test and Levene's test for equality of variances, respectively. If the normality and homogeneity assumptions were met, a one‐way analysis of variance (ANOVA) was used, and the Kruskal–Wallis test otherwise. Two‐by‐two comparisons were performed with the post hoc Tukey–Kramer test for ANOVA results, or the Conover–Iman test for the Kruskal–Wallis results. As a secondary analysis, the presence of aura was included as an additional covariate considering that previous studies have reported differences between patients with migraine with and without aura (Messina et al., 2013; Szabó et al., 2017).

For each couple of gray matter and structural connectivity features, results were corrected for multiple comparisons using the Benjamini–Hochberg false discovery rate method (Benjamini & Hochberg, 1995). The total number of comparisons, excluding the post hoc tests, was equal to the number of independent components multiplied by the number of modalities (two).

The level of statistical significance was set at *p* < .05.

#### 2.5.2 Source components

To identify the regions and connections which were altered in a specific component, the Z‐scores distributions were analyzed. The regions or connections that contained positive and negative outliers of the distribution, that is, absolute values larger than three scaled median absolute deviations from the median, were considered respectively as strengthened and weakened regions or connections.

In the analysis of source components from structural connectivity, diverse pairs of connected regions should be identified. Some of these pairs could share one of the connected regions. In this situation, the identified connections would represent a strengthened or weakened “network", that is, a group of interconnected regions with altered structural connectivity.

#### 2.5.3 Correlation analysis

Correlations of mixing coefficients from components and modalities with significant differences between groups and clinical parameters were obtained to study the potential impact of the discovered patterns in migraine symptoms. These clinical parameters were the duration of disease, time from onset of CM in these patients, and headache and migraine frequency. The Spearman's rank correlation coefficient was the employed measure.

## 3 RESULTS

### 3.1 Subject characteristics

Table 1 shows the clinical and demographic characteristics of the subjects. There were no significant differences in age and gender between the subjects from different groups. With respect to EM, CM patients had a longer duration of disease, higher headache and migraine frequency, higher proportion of patients overusing medication, and lower proportion of aura. Further details can be found in Planchuelo‐Gómez, et al. (2020a, 2020b).

**TABLE 1:** Clinical and demographic characteristics of HC, EM, and CM

	HC (*n* = 50)	EM (*n* = 54)	CM (*n* = 56)	Statistical test
Gender, male/female	11/39 (22/78%)	9/45 (17/83%)	6/50 (11/89%)	*χ* ^2^_(2,*N* = 160)_ = 2.48, *p* = .29^a^
Age (years)	36.1 ± 13.2	37.1 ± 8.2	38.1 ± 8.7	*χ* ^2^ (2) = 2.85, *p* = .24^b^
Duration of migraine history (years)		14.1 ± 11.1	19.6 ± 10.4	*t*_(108)_ = −2.7, *p* = .008^c^
Time from onset of chronic migraine (months)			24.5 ± 32.9	
Headache frequency (days/month)		3.6 ± 1.9	23.3 ± 6.3	*U* = 44.0, *p* < .001^d^
Migraine frequency (days/month)		3.6 ± 1.9	13.9 ± 6.9	*U* = 108.5, *p* < .001^d^
Overusing medication		0 (0%)	42 (75%)	*p* < .001^e^
Aura		9 (17%)	1 (2%)	*p* = .007^e^

*Note:* Data are expressed as means ± *SD*.

Abbreviations: CM, chronic migraine; EM, episodic migraine; HC, healthy controls.

^**a**^ Chi‐square test.

^b^ Kruskal–Wallis test.

^c^ Two‐tailed, unpaired Student's *t* test.

^d^ Mann–Whitney *U* test.

^e^ Fisher's exact test.

### 3.2 Components with significant differences

Three components with significant differences were found:

- One multimodal component (IC1) was identified in the analysis of gray matter cortical curvature and structural connectivity.
- One modal‐specific component was identified in the analysis of cortical thickness and structural connectivity (IC5, structural connectivity specific component)
- One modal‐specific component was identified in the analysis of gray matter volume and structural connectivity (IC3, structural connectivity specific component).

No significant differences were found in the analysis of surface area and connectivity after correction for multiple comparisons (four independent components were obtained). The comparisons of each component with significant differences between CM, EM, and HC are presented in three sections.

#### 3.2.1 Cortical curvature and structural connectivity

Two independent components were obtained. The number of retained components was two in the case of the cortical curvature, and 22 for the structural connectivity. A joint component was identified (IC1), indicating association between the changes in curvature and in structural connectivity.

According to the values of the mixing coefficients and IC1 Z‐scores, EM patients presented higher curvature values in the bilateral frontal pole in comparison to HC (mixing coefficients; *F*_2,157_ = 5.26, corrected *p* = .012; Tukey–Kramer test *p* = .004). Also, CM patients showed higher gray matter curvature in the left rostral anterior cingulate cortex with respect to controls, but statistical significance was not reached in this comparison (Tukey–Kramer test *p* = .064).

Based on the connectivity mixing coefficients and IC1 Z‐scores, significant higher connectivity was observed in EM compared to HC (mixing coefficients; *F*_2,157_ = 6.53, corrected *p* = .008; Tukey–Kramer test *p* = .001), and in CM compared to HC (Tukey–Kramer test *p* = .044). In EM compared to HC, connections between regions from the parietal cortex, especially in the superior area, were reinforced in patients. In CM compared to HC, connections between the cingulate cortex and frontal regions, and connections between the hippocampus and regions from the temporal cortex, were strengthened. In both patient groups, strengthened connections between orbitofrontal regions, the putamen and the insula were observed. Connections between orbitofrontal regions and the caudate nucleus were decayed in CM with respect to EM. Reinforced connections between the hippocampus, the putamen and the insula were found exclusively in CM. Following IC1 sources from the three groups, connections of the pars orbitalis and triangularis with the rostral middle frontal gyrus were only detected in controls. Detailed strengthened and decayed networks are shown in Supplementary Tables 1–4, indicated as connections with positive and negative outliers (from IC1 sources), respectively.

**FIGURE 2:**
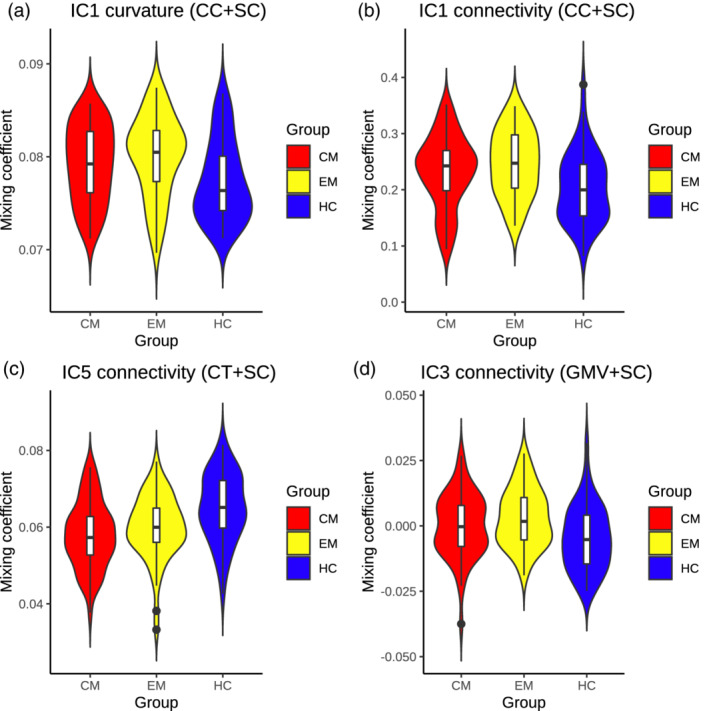
Violin and box plots illustrating the distribution of the mixing coefficient values on each group in the comparisons with significant differences. Significant higher mixing coefficients in both patient groups compared to controls in (a) mean cortical curvature (CC) is higher in the patients in the regions with positive Z‐scores. The same significant trend is shown in (b), and the interpretation is the same but with structural connectivity (SC). Significant lower coefficients in both patient groups with respect to controls are shown in (c), which means that SC is debilitated in patients than in controls in the connections with positive Z‐scores. (d) Significant higher coefficients in episodic migraine (EM) in comparison to healthy control (HC), with the same interpretation as (b) for positive Z‐scores and mixing coefficients, and the opposite trend for negative coefficients and positive Z‐scores (or positive coefficients and negative Z‐scores). CT, cortical thickness; GMV, gray matter volume

Mixing coefficients of IC1 of both modalities were significantly correlated (Pearson's *r* = .579, *p* < .001). IC1 mixing coefficients violin plots from both modalities can be seen in Figure 2a,b.

**FIGURE 3:**
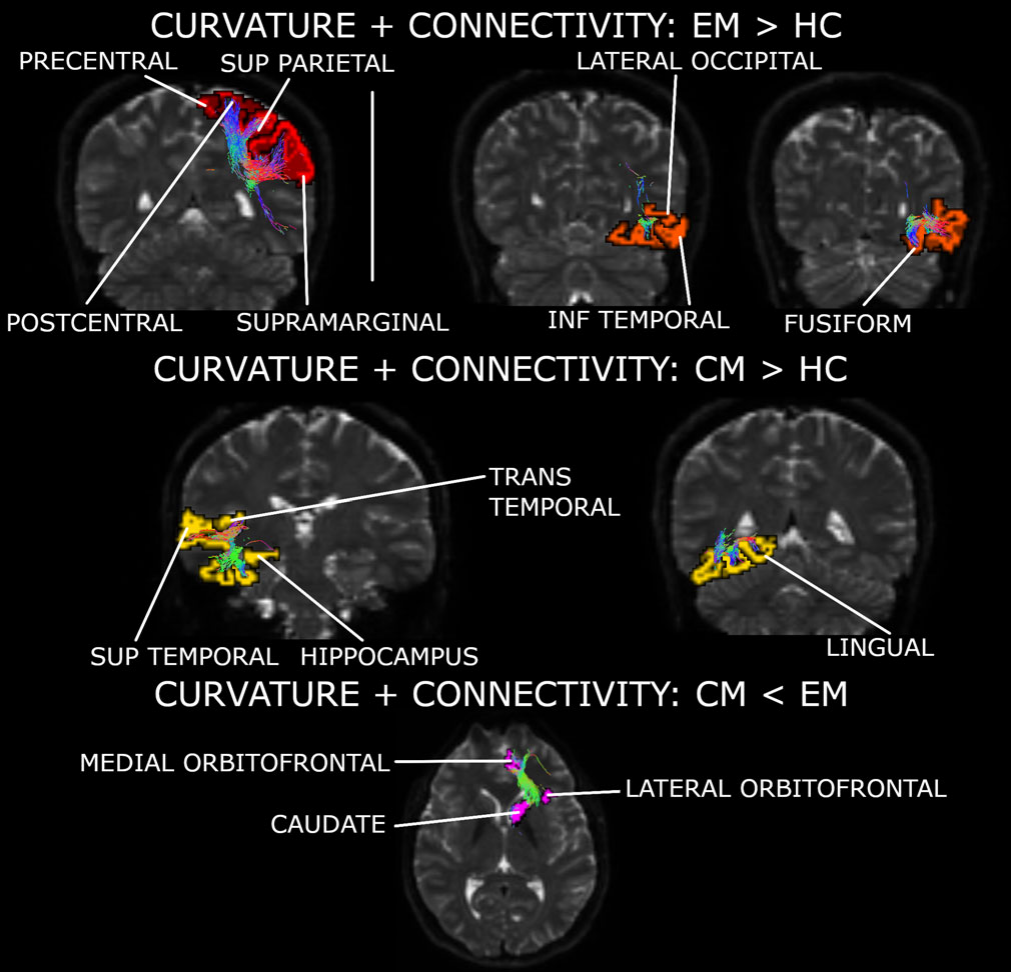
Major networks found for the first independent component (fusion of curvature and connectivity). Two networks were strengthened in episodic migraine (EM) compared to healthy control (HC), with involved regions from central, parietal, temporal and occipital areas. One network was reinforced in chronic migraine (CM) compared to HC, with involved regions from the temporal cortex and the hippocampus. The network involving regions from the orbitofrontal cortex and the caudate nucleus was debilitated in CM with respect to EM. Regions represented in a hemisphere may be associated with a specific or both hemispheres (more details can be found in Supplementary File 3). INF, inferior; SUP, superior

A summary of these networks can be found in Figure 3 and Supplementary Figure 1. The previous significant results suffered no variations when adjusting them by the effect of the presence of aura.

#### 3.2.2 Cortical thickness and structural connectivity

Six independent components were obtained. The number of components was six in the case of the cortical thickness, and 22 for the structural connectivity. A structural connectivity modal‐specific component was identified (IC5). No significant differences were detected in any thickness component.

Following mixing coefficients values and IC5 Z‐scores, lower structural connectivity values were found in EM compared to HC (mixing coefficients; *F*_2,157_ = 9.41, corrected *p* = .002; Tukey–Kramer test *p* = .007), and in CM compared to HC (Tukey–Kramer test *p* < .001).

In both EM and CM patient groups, connections between diverse gyri within the temporal cortex and fusiform gyrus were weakened with respect to controls. Connections between regions from the parietal lobule, precuneus, cuneus, pericalcarine cortex, and supramarginal gyrus were also debilitated in patients compared to controls. Weakening in EM with respect to controls was also observed in connections between frontal regions, connections which were not found in the IC5 specific CM source, reflecting a possible weakening in CM compared to EM.

Observing IC5 sources from the three groups, exclusive debilitated connections were found in CM between the superior temporal gyrus, isthmus cingulate cortex, thalamus, and hippocampus. Following IC5 sources from the three groups, strengthened connections of the pars orbitalis and triangularis with frontal regions were only found in controls.

Reinforced connections in the patient groups with respect to the controls were found between the insula and the temporal cortex.

**FIGURE 4:**
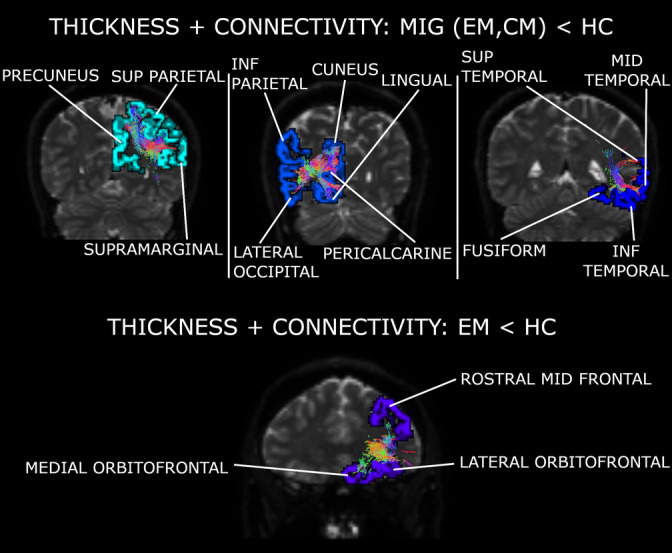
Major networks found for the fifth independent component (fusion of thickness and connectivity). Three networks were debilitated in both migraine groups in comparison to controls. These networks included regions from the parietal, occipital, and temporal lobes. One network including regions from the frontal cortex was damaged in chronic migraine (CM) compared to healthy control (HC). Regions represented in a hemisphere may be associated with a specific or both hemispheres (more details can be found in Supplementary File 3). INF, inferior; MID, middle; MIG, migraine (results from episodic migraine [EM] and CM); SUP, superior

IC5 structural connectivity mixing coefficients can be found in Figure 2c. Detailed strengthened and decayed networks are shown in Supplementary Tables 5–9, indicated as connections with positive and negative outliers (from IC5 sources) respectively. A summary of the networks from this section can be found in Figure 4 and Supplementary Figure 2. The previous significant results suffered no variations when adjusting them by the effect of the presence of aura.

#### 3.2.3 Gray matter volume and structural connectivity

Five independent components were obtained. The number of retained components was five in the case of the gray matter volume, and 21 for the structural connectivity. A structural connectivity, modal‐specific component was identified (IC3). No significant differences were detected in any volume component.

In this analysis, differences between groups were more related to differences between IC3 sources from the three groups, that is, different sign of Z‐scores in the patients compared to HC, than to higher or lower weight (mixing coefficients) of the connections.

Structural connectivity was strengthened in patients with respect to controls in the network composed of the thalamus, caudate nucleus, lateral orbitofrontal cortex, precentral gyrus, putamen, rostral anterior cingulate cortex, insula (only in EM), and hippocampus (only in CM). In EM with respect to controls, connectivity was also reinforced in the connection of the superior parietal cortex with the postcentral gyrus and paracentral lobule.

Migraine patients presented weakened structural connectivity in comparison to controls in networks mentioned in the previous cases, for example, the connections within the temporal and the frontal cortex.

EM patients mixing coefficients were significantly higher in comparison to controls (*F*_2,157_ = 6.01, corrected *p* = .031; Tukey–Kramer test *p* = .002). No significant differences were found in comparisons with CM patients mixing coefficients.

**FIGURE 5:**
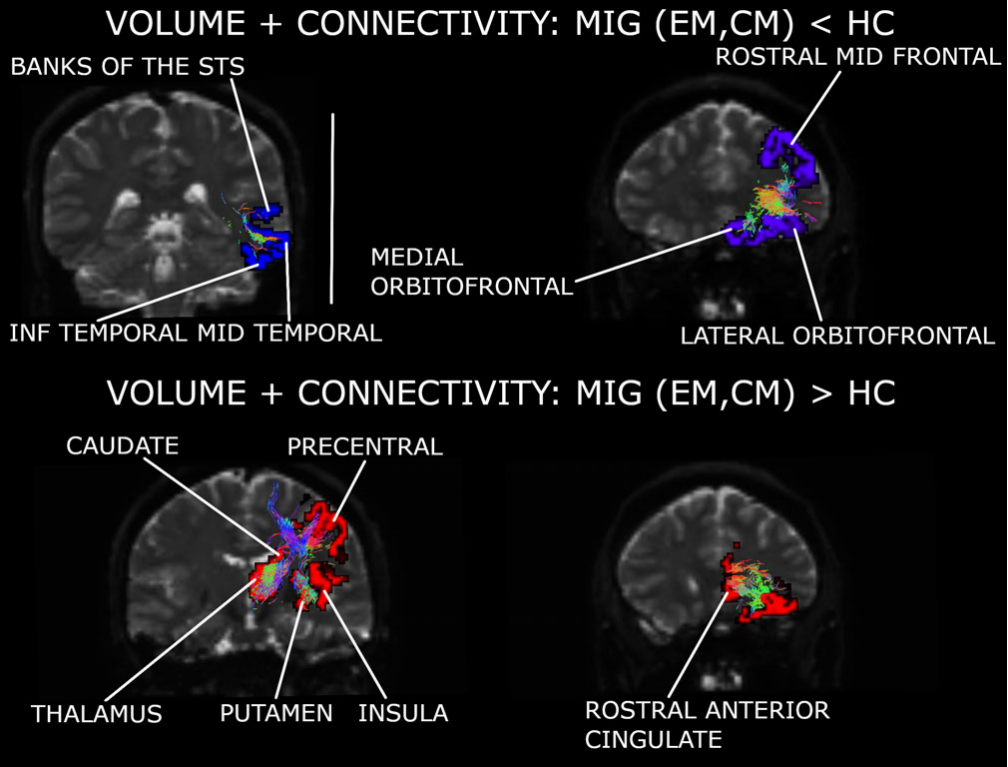
Major networks found for the third independent component (fusion of gray matter volume and connectivity). Two networks were debilitated in both migraine groups compared to controls. These regions included areas from the temporal and frontal lobes. One network was strengthened in both migraine groups with respect to controls. This network included subcortical regions, the insula, one region from the frontal cortex and the cingulate gyrus and the precentral gyrus. Regions represented in a hemisphere may be associated with a specific or both hemispheres (more details can be found in Supplementary File 3). INF, inferior; MID, middle; MIG, migraine (results from episodic migraine [EM] and chronic migraine [CM]); STS, superior temporal sulcus

IC3 structural connectivity mixing coefficients can be found in Figure 2d. Detailed strengthened and decayed networks are shown in Supplementary Tables 10–15, indicated as connections with positive and negative outliers (from IC3 sources), respectively. A summary of the networks from this section can be found in Figure 5 and Supplementary Figures 3 and 4. The previous significant results suffered no variations when adjusting them by the effect of the presence of aura.

### 3.3 Relation between gray matter morphometric features

From the simultaneous analysis of curvature and thickness (*F*_2,157_ = 5.36, corrected *p* = .022; Tukey–Kramer test EM vs. HC *p* = .004), and also that of curvature and area (*F*_2,157_ = 4.41, corrected *p* = .046; Tukey–Kramer test EM vs. HC *p* = .010), a significant curvature modal‐specific component was obtained. Results were similar to those from the analysis of curvature and structural connectivity. Patients with EM showed higher curvature compared to HC in regions such as the frontal and temporal poles. Higher curvature (Z‐score positive outlier) was found in the left rostral anterior cingulate cortex in patients with CM, but no significant results related to mixing coefficients were identified in CM.

A significant area modal‐specific component in the analysis of curvature and area was found. This component showed that surface was higher in EM compared to CM (*F*_2,157_ = 3.86, corrected *p* = .046; Tukey–Kramer test *p* = .027). No specific region (outlier) was identified in the EM source, but the associated Z‐scores from the bilateral superior frontal gyrus, where specific regions were found in EM, the right inferior parietal cortex, and the right middle frontal gyrus were higher than two.

No significant results were identified for the cortical thickness.

### 3.4 Correlation analysis

No significant correlation between mixing coefficients and clinical parameters was found.

## 4 DISCUSSION

This study introduces two main novel elements. On the one hand, it is the first study to compare migraine patients and controls with an integrated multimodal approach, mCCA‐jICA. On the other hand, mCCA‐jICA was employed for the first time to analyze simultaneously features from structural connectivity (connectomics) and gray matter morphometry, instead of directly using maps from MRI (e.g., DTI) or segmented images (e.g., gray matter). This new approach allowed us to identify structural network differences in EM and CM with respect to controls, and in CM compared with EM. More importantly, cortical curvature differences between EM and HC were detected and found to be related to the structural connectivity.

### 4.1 Gray matter morphometry

We found significant higher curvature in EM compared to HC, and increased expression in the rostral anterior cingulate gyrus in CM. The increased curvature in both groups of migraine patients is in line with our previous results with the same sample (Planchuelo‐Gómez, García‐Azorín, Guerrero, Rodríguez, et al., 2020) but, interestingly, the regions found with higher or reinforced curvature in migraine in this study were different from our previous study analyzing gray matter morphometry. While multimodal analysis may be able to uncover new patterns, this does not exclude the need for single‐modality conventional analyses.

Although both studies follow the same general trends (increased curvature in migraine), the differences between them may reflect that cortical curvature changes could be related to two different but related mechanisms. The first process, suggested by the results of this study, would be influenced by increased structural connectivity between gray matter regions. The second would be related to white matter atrophy, as suggested previously in multiple sclerosis and schizophrenia studies analyzing curvature and DTI measures (Deppe et al., 2014; Lubeiro et al., 2017).

The differences between the two studies with the same sample may come from the mathematical model employed in this study. On the one hand, the curvature values assessed in the direct comparison (Planchuelo‐Gómez, García‐Azorín, Guerrero, Rodríguez, et al., 2020) reflect the effect of all the biological processes or external factors which might influence the brain structure. On the other hand, the results from this study indicate the specific enhanced features which are related to another specific mechanism, in this case, the association between cortical curvature and structural connectivity. Thus, the methodology employed in this study is able to detect the relationships between individual biological processes or factors.

No joint components were found using cortical thickness, surface area and gray matter volume as morphometric features. In contrast, functional connectivity alterations have been found in regions where gray matter volume loss was identified in migraine (Burke et al., 2020). In that study, positive and negative functional connections between these regions, extracted from (Jia & Yu, 2017), and other regions were found. Considering also the results from (Burke et al., 2020), it may be hypothesized that, at least in migraine, cortical curvature changes would be related to changes in structural connectivity, and even white matter structure, while changes in gray matter volume would be more related to changes in functional connectivity. The possible association of gray matter morphometry and structural connectivity with functional connectivity should be studied in the future, especially considering that no clear relationship between curvature, thickness and area was found according to our results.

### 4.2 Structural connectivity

**FIGURE 6:**
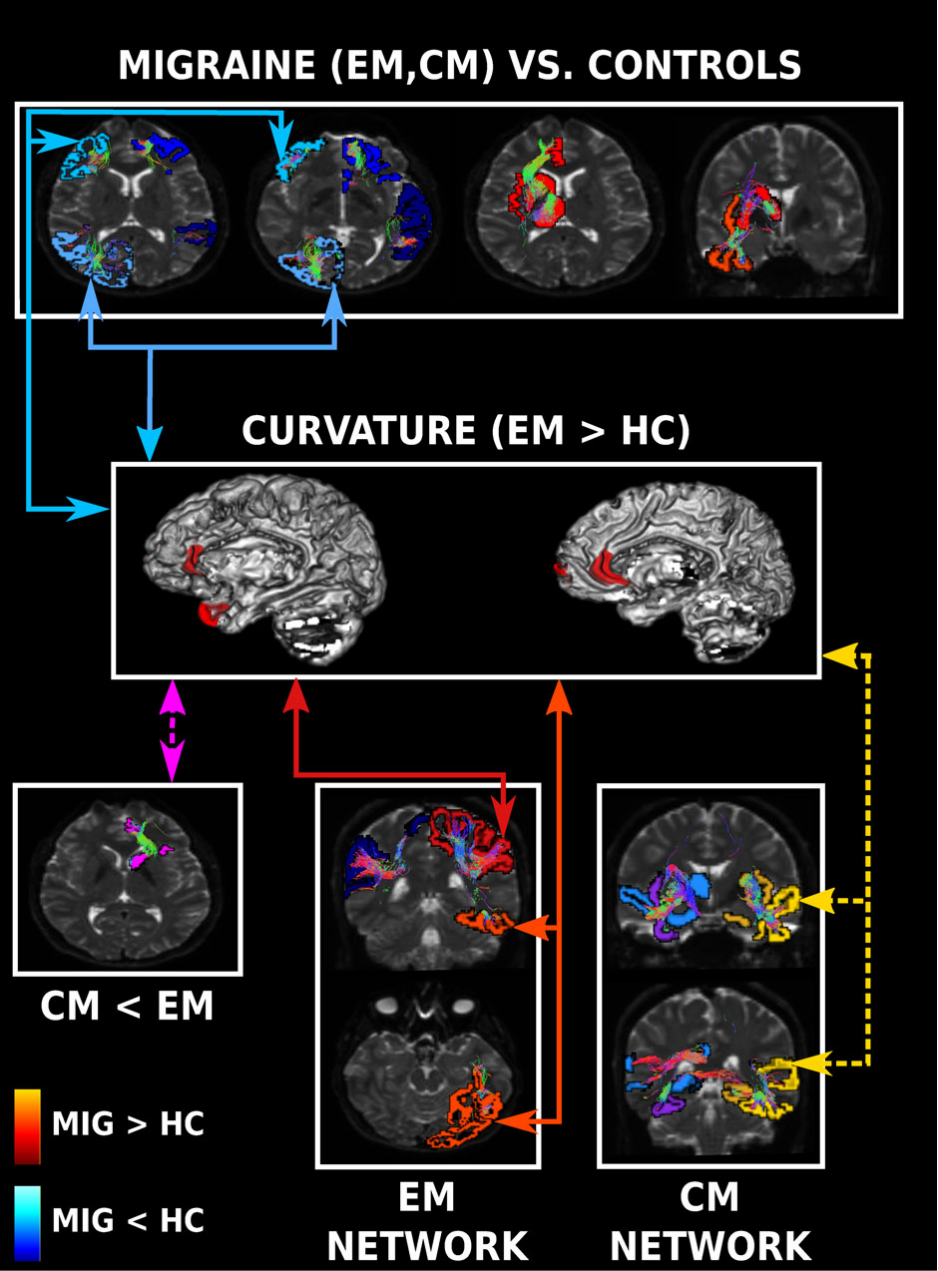
Summary of the identified structural networks and gray matter morphometry differences. Regions and arrows in blue colors represent weakened structural connectivity in migraine, while red, orange, and yellow represent strengthened connectivity and cortical curvature in migraine. Regions and arrow in magenta are used to show differences between chronic migraine (CM) and episodic migraine (EM). The arrows represent changes found simultaneously in cortical curvature and structural connectivity, using dashed arrows when changes were not directly related to comparisons between EM and healthy control (HC), but in joint components related to cortical curvature. Regions represented in a hemisphere are illustrative and do not reflect changes found in a specific hemisphere. MIG, migraine (results from EM and CM)

Three main structural connectivity patterns were obtained with the joint modal analysis, which are summarized in Table 2 and Figure 6. The first and second identified patterns were weakened and strengthened connectivity in migraine patients compared to controls, and the third pattern was related to specific networks expressed in EM and CM.

**TABLE 2:** Identified structural networks with differences between HC, EM, and CM patients

CM, EM < HC	1. Lateral orbitofrontal – Medial orbitofrontal – **Rostral middle frontal**
	2. **Rostral middle frontal** – Pars orbitalis – Pars triangularis
	3. Banks of the superior temporal sulcus – Inferior temporal – Middle temporal – Fusiform – Superior temporal
	4. Cuneus – Lingual – Lateral occipital – **Inferior parietal**
	5. **Inferior parietal** – Precuneus – Supramarginal – Superior parietal
CM, EM > HC	1. Rostral anterior cingulate – Thalamus – Caudate – Putamen – Lateral orbitofrontal – Medial orbitofrontal – **Insula**
	2. **Insula** – Fusiform – Inferior temporal
Worsened in EM	1. Postcentral – Supramarginal – Inferior parietal
Enhanced in EM	1. Superior parietal – Supramarginal – Precentral – Postcentral – Paracentral
	2. Fusiform – Inferior temporal – Lateral occipital
Worsened in CM	1. Insula – Pallidum – Fusiform
	2. Thalamus – Hippocampus – Superior temporal – Isthmus cingulate
CM < EM	1. Lateral orbitofrontal – Medial orbitofrontal – Caudate
Enhanced in CM	1. Insula – Putamen – Parahippocampal – **Hippocampus**
	2. **Hippocampus** – Fusiform – Inferior temporal – Superior temporal – Lingual – Transverse temporal

*Note:* Regions in bold are implied in two different networks with the same trend. Underlined regions are implied in diverse networks with reinforced and debilitated connectivity in migraine, EM or CM.

Abbreviations: CM, chronic migraine; EM, episodic migraine; HC, healthy controls.

#### 4.2.1 Weakened connectivity in migraine

Several networks were weakened in migraine (both EM and CM) in comparison to HC. These networks contained in most of the cases regions within each of the four lobes. The same trend in connections within the temporal and the frontal lobes has been detected in a connectomics study (Planchuelo‐Gómez, et al., 2020a), but, in the present study, the detection of these networks was better defined thanks to the multimodal analysis.

One of the regions involved in debilitated networks was the inferior parietal cortex. Increased blood oxygen level‐dependent (BOLD) signal using functional MRI (fMRI) has been found in the inferior parietal cortex in migraine with aura (Hougaard et al., 2014). Thickening has also been found in the inferior parietal cortex in migraine with aura compared to HC and migraine without aura (Messina et al., 2013). The inferior parietal lobe is involved in visuotemporal attention (Shapiro, Hillstrom, & Husain, 2002). We did not detect simultaneous thinning or thickening related to structural connectivity alterations. Therefore, taking into account the fMRI results, weakened structural connectivity could be related to strengthened functional connectivity in migraine. This result is unusual, although coexistence of high functional connectivity and low structural connectivity has been found in healthy subjects (Koch, Norris, & Hund‐Georgiadis, 2002), and inverse correlation between both types of connectivity has been identified in multiple sclerosis (Lowe et al., 2008). Further studies are needed to assess the relationship between structural and functional connectivity.

Higher cortical thickness has been reported in migraine in comparison to HC in the middle frontal gyrus (Messina et al., 2013). Increased BOLD signal has been found in the inferior frontal cortex in migraine (Hougaard et al., 2014), an area connected with the rostral middle frontal gyrus in our results (debilitated connectivity in migraine). The anterior part of the frontal lobe takes part in executive functions (Koechlin & Hyafil, 2007). The frontal region results from the literature were similar to those mentioned for the inferior parietal cortex. The concordance of these results is in line with the previous hypothesis about opposite trends between structural and functional connectivity.

#### 4.2.2 Strengthened connectivity in migraine

Subcortical regions and the insula were involved in networks found to be strengthened in both EM and CM with respect to controls. The same trend has been reported previously (Planchuelo‐Gómez, et al., 2020a), but with some differences in the connections with significant differences. The insula was involved in the two identified networks reinforced in EM and CM. Thinning and gray matter volume loss have been found in migraine compared to HC (Messina et al., 2013; Planchuelo‐Gómez, García‐Azorín, Guerrero, Rodríguez, et al., 2020). Positive functional connections between regions with gray matter volume loss and the insula have been identified (Burke et al., 2020), but involved regions were different with respect to those included in Table 2. The insula has been reported to be involved in many functional alterations in migraine, processing afferent and efferent information (Borsook et al., 2016). Hence, our results would reinforce the idea of the key role of the insula in migraine not only in functional connectivity, but also in strengthened structural connectivity.

One of the identified strengthened networks in migraine included the thalamus, the caudate nucleus and the putamen. Lower volume in thalamic nuclei has been found in migraine compared to HC (Magon et al., 2015). In a review, the thalamus has been reported to be involved in dysfunctional pain modulation and processing, allodynia, central sensitization, and photophobia in migraine (Younis, Hougaard, Noseda, & Ashina, 2019). Reduced volume in the caudate (Yuan et al., 2013) and in the putamen (Petrusic, Dakovic, & Zidverc‐Trajkovic, 2019) has been reported in migraine compared to HC, and also dysfunctional connectivity in the putamen, suggesting that the putamen is a key region in the integration of information in migraine (Zhao et al., 2014). In the case of CM, higher gray matter volume compared to controls has been identified in the putamen (Neeb et al., 2017), a result possibly related to one of the enhanced networks in CM from our results, which was composed of the insula, putamen, parahippocampal gyrus, and hippocampus. The caudate nucleus may play an important role in the modulation of the pain experience (Wunderlich et al., 2011). Following the possible opposite trends between the functional and the structural connectivity mentioned before in the connections between cortical regions, the results with the subcortical regions were consistent, but with higher structural and lower functional connectivity instead. Some connected regions with increased structural connectivity in our study have shown increased functional connectivity during migraine attacks (Amin et al., 2018). Thus, the structural connectivity in migraine may reflect the networks which are hyper‐ and hypoactive in ictal state. In interictal state, the functional connectivity could be counterbalanced compared to the ictal state, while the brain may suffer structural changes as an adaptation to attacks.

An interesting situation with opposite trends for the structural connectivity was observed in the orbitofrontal cortex. The orbitofrontal cortex was involved in a weakened network in migraine, within the frontal lobe, and a strengthened network, in connections with the insula and the putamen. Reduced gray matter volume and increased functional connectivity with the dorsal anterior cingulate cortex have been found in the orbitofrontal cortex in migraine patients (Jin et al., 2013; Kim et al., 2008). Lower gray matter volume in the orbitofrontal cortex has been related to poor response to treatment in migraine (Jia & Yu, 2017).

#### 4.2.3 Structural networks in EM and CM

The connections with the orbitofrontal cortex played a role not only in the identification of differences between migraine patients and HC, but also between CM and EM. The only network with clear differences from the sources between CM and EM was composed of lateral and medial orbitofrontal regions from the Desikan–Killiany atlas and the caudate nucleus. A neuropsychological evaluation study has reported worse orbitofrontal task performance in CM with respect to EM and HC and associated this baseline performance with negative outcome after one year follow‐up (Gómez‐Beldarrain, Carrasco, Bilbao, & García‐Moncó, 2011). In an fMRI study, the caudate nucleus presented lower response to noxious stimulation in high‐frequency EM in comparison to low‐frequency EM, and also lower functional connectivity with the insula and higher gray matter volume (Maleki et al., 2011). These previous results and our findings suggest that the structural and functional connectivity of the orbitofrontal cortex with pain processing regions such as the caudate nucleus and the insula may play a key role in the effect of the treatment and progression of migraine.

About the exclusive networks found for EM, the weakened network was composed of regions from the parietal lobe, a result in line with the comparison between migraine patients and HC. The strengthened networks in EM were composed of regions from two lobes. One of these networks included regions from the parietal and frontal lobes, and the other one from the temporal and occipital lobes. This finding suggests that connections between regions from different lobes, possibly integrating diverse aspects related to the functions affected by the pain experience, may be reinforced in EM compared to controls.

The hippocampus was the region which was highlighted only in the CM exclusive networks. In the two CM reinforced networks, the hippocampus was one of the regions involved. In a review, the hippocampus has been reported as a key region related to migraine prognosis, associating a smaller volume and higher values of graph theory measures from DTI with a worse prognosis (Liu, Chou, & Chen, 2018). Thus, the hippocampus structural connectivity with regions from the inferior temporal lobe or the insula and putamen seems to be a possible CM biomarker.

The hippocampus was also involved in a weakened network in CM that presented connections with the thalamus and the superior temporal gyrus. Another debilitated network in migraine contained the insula, the pallidum and the fusiform gyrus. In high‐frequency EM, higher functional connectivity in the connection between the insula and the pallidum has been observed in comparison to low‐frequency EM (Maleki et al., 2011). These results may imply that the hippocampus may not only participate as an active structural connection center in CM, but also may be involved in damaged structural connections with the thalamus, an important pain processing region.

### 4.3 Novel perspective of the multimodal fusion analysis

Throughout the discussion section, we have hypothesized a possible inverse relationship between structural and functional connectivity. These possible opposite trends may show a maladaptation process to counterbalance strengthened or weakened structural connectivity. Thus, multimodal fusion analysis may be helpful to uncover new relationships between brain structure and function and raise new hypotheses.

Sophisticated fusion methods can be useful for purposes beyond simply obtaining replicated results from direct comparison of MRI features. Methods such as mCCA‐jICA allow to capture complex covariance and relationships between specific modalities and to find joint alteration patterns between diverse groups of interest. Therefore, the mCCA‐jICA method can identify additional alterations which are complementary to the single‐modality analysis and find alteration patterns related to simultaneous changes in brain structure and activity. The fusion methods may help to better understand and integrate findings from diverse modalities.

### 4.4 Limitations

This study has some limitations. Concerning the dataset, and as mentioned in our previous studies with the same sample (Planchuelo‐Gómez, et al., 2020a, 2020b), white matter hyperintensities could not be assessed due to the lack of T2 or T2‐FLAIR MRI sequences, and there could be a certain bias in the CM patients due to the great percentage (75%) of medication overuse patients. Additionally, we controlled that the patients suffered no migraine attacks during the 24 hr prior to the MRI acquisition, but there was no control for the next 24 hr (or more). Therefore, some patients could possibly have been scanned in the prodromal phase of migraine, instead of the interictal phase, which might have influenced the results. With regard to the streamline count as a connectivity measure, although its use has been sometimes controversial, current trends consider it to be an acceptable metric for connectivity as long as it is based on appropriate tractography methods, such as the anatomically‐constrained tracking algorithm that we employed (Yeh, Jones, Liang, Descoteaux, & Connelly, 2020). About limitations regarding specifically this study, no fMRI data were available to confirm the hypothesis of the inverse relationship between structural and functional connectivity, and thus we could only associate our structural connectivity findings to results from the literature. With respect to the sources used to identify the networks or specific regions on the independent components, the criterion to highlight them was not based on statistical inference, but only on Z‐score outliers from independent components with significant differences between groups.

## 5 CONCLUSION

Our findings suggest that, in migraine patients, structural networks composed of cortical regions within each lobe are weakened, while networks with subcortical or pain processing regions such as the insula are strengthened. In migraine, cortical curvature changes are related to structural connectivity alterations, which may be also affected by functional connectivity, while cortical thickness, surface area, and gray matter volume changes may be associated with the functional activity variations. The strengthened and/or weakened connections with the hippocampus and damaged structural connectivity between the orbitofrontal cortex and the caudate nucleus may be biomarkers for CM. Reinforced connections between the central sulcus and regions from the superior parietal cortex were found in EM. Fusion methods such as mCCA‐jICA allow to assess relationships between multiple modalities, providing additional insights and results. Future multimodal studies analyzing the possible inverse relationship between structural and functional connectivity, and the relationship between gray and white matter structure and activity in migraine patients, need to be performed.

## CONFLICT OF INTEREST

The authors declare no conflict of interests.

## ETHICS STATEMENT

The local Ethics Committee of Hospital Clínico Universitario de Valladolid approved the study (PI:14‐197).

## PATIENT CONSENT STATEMENT

All participants read and signed a written consent form prior to their participation.

## Supporting information


**Appendix** **S1.** Supporting Information.

**Appendix** **S2.** Supporting Information.

**Appendix** **S3.** Supporting Information.

**Appendix** **S4.** Supporting Information.

## Data Availability

The datasets that support the findings of this study are available from the corresponding author, upon reasonable request.
