# Supplementary material for: Multimodal fusion analysis of structural connectivity and gray matter morphology in migraine
Source: Hum Brain Mapp. 2020 Dec 8;42(4):908–21. doi: 10.1002/hbm.25267 (PMC7856653; doi:10.1002/hbm.25267)
Supplement: Supplementary file 1 — Appendix S1. Supporting Information. [file HBM-42-908-s001.pdf]

Supplementary File 1

In this file, diverse aspects from previous studies not developed in the main manuscript are explained.

Participants

The inclusion and exclusion criteria for patients and controls are described here from (Planchuelo-Gómez, García-Azorín, Guerrero, Aja-Fernández, et al., 2020a, 2020b).

Migraine patients were screened and recruited at the headache unit at the Hospital Clínico Universitario de Valladolid (Valladolid, Spain), being classified as Episodic Migraine (EM) or Chronic Migraine (CM) following the third edition of the International Classification of Headache Disorders (ICHD and ICHD-3 beta) aged between 18 and 60 (Headache Classification Committee of the International Headache Society, 2013, 2018). Patients were clinically stable the three months before the visit, preventive treatment naïve and with migraine onset before the age of 50. All patients agreed to participate in the study and signed an Informed Consent.

Patients were excluded if they suffered headache on 10-14 days per month (to avoid confusion between high frequency EM and CM), non-craniofacial painful conditions occurring 10 or more days per month apart from migraine, known major psychiatric diseases and other neurological diseases or headache disorders. Drug and substance abuse, pregnancy and childbearing were other exclusion criteria.

MRI acquisition details

Migraine patients were scanned between one and two weeks after the clinical visit if the last migraine attack happened at least 24 hours before the acquisition. The first acquisition was the T1-weighted, being followed by the diffusion-weighted scan. The images were

obtained between May 2014 and July 2018. Total acquisition time for a single subject was around 18 minutes.

Morphometry processing pipeline

A detailed summary of the processing pipeline explained in (Planchuelo-Gómez, García-Azorín, Guerrero, Rodríguez, et al., 2020) is shown.

The first step was to extract the non-brain tissue from the T1-weighted images. The FreeSurfer gray matter parcellation was performed afterwards. FreeSurfer parcellation includes skull stripping, automated Talairach transformation, segmentation of subcortical grey and white matter, intensity normalization, grey-white matter boundary tessellation and surface deformation. The results from the parcellation were examined individually. Cortical curvature, cortical thickness, surface area and gray matter volume were extracted for the 68 cortical regions from the Desikan-Killiany atlas (Desikan et al., 2006). The gray matter volume was also extracted for the 16 subcortical regions from the same atlas. These 84 cortical and subcortical regions were used as regions in the structural connectivity matrices analyzed in this study.

Structural connectivity processing pipeline

A detailed summary of the processing pipeline explained in (Planchuelo-Gómez, García-Azorín, Guerrero, Aja-Fernández, et al., 2020a) is shown.

Apart from the FreeSurfer parcellation, diffusion-weighted images were preprocessed. The first step was to denoise the images. The next steps were corrections for eddy currents, motion and B1 field inhomogeneity. From the preprocessed images, a whole brain mask was obtained. Separately, five-tissue-type segmented images were obtained from the T1-weighted images and the cortical parcellation in each subject.

The fiber orientation distributions (FOD) were estimated using the diffusion images to perform tractography, after the estimation of the response function. Spherical deconvolution was employed in the estimation of the FOD. The diffusion mask, the five-tissue-type image, registered previously to the diffusion space for each subject, and the FOD were used to perform anatomically-constrained tractography on each subject, computing 10 million streamlines.

From the tractography, structural connectivity matrices were acquired. The gray matter cortical parcellation regions were included in the structural connectivity matrix from each subject.

References

Desikan, R. S., Ségonne, F., Fischl, B., Quinn, B. T., Dickerson, B. C., Blacker, D., Buckner, R. L., Dale, A. M., Maguire, R. P., Hyman, B. T., Albert, M. S., & Killiany, R. J. (2006). An automated labeling system for subdividing the human cerebral cortex on MRI scans into gyral based regions of interest. *NeuroImage*, *31*(3), 968–980. https://doi.org/10.1016/j.neuroimage.2006.01.021

Headache Classification Committee of the International Headache Society. (2013). The International Classification of Headache Disorders, 3rd edition (beta version). *Cephalalgia*, *33*(9), 629–808. https://doi.org/10.1177/0333102413485658

Headache Classification Committee of the International Headache Society. (2018). The International Classification of Headache Disorders, 3rd edition. *Cephalalgia*, *38*(1), 1–211. https://doi.org/10.1177/0333102413485658

Planchuelo-Gómez, Á., García-Azorín, D., Guerrero, Á. L., Aja-Fernández, S., Rodríguez, M., & de Luis-García, R. (2020a). Structural connectivity alterations in chronic and episodic migraine: A diffusion magnetic resonance imaging connectomics study. *Cephalalgia*, *40*(4), 367–383. https://doi.org/10.1177/0333102419885392

Planchuelo-Gómez, Á., García-Azorín, D., Guerrero, Á. L., Aja-Fernández, S., Rodríguez, M., & de Luis-García, R. (2020b). White matter changes in chronic and episodic migraine: a diffusion tensor imaging study. *J Headache Pain*, *21*(1), 1. https://doi.org/10.1186/s10194-019-1071-3

Planchuelo-Gómez, Á., García-Azorín, D., Guerrero, Á. L., Rodríguez, M., Aja-Fernández, S., & de Luis-García, R. (2020). Grey matter structural alterations in chronic and episodic migraine: a morphometric magnetic resonance imaging study.

Pain Medicine. https://doi.org/10.1093/pm/pnaa271
