# Supplementary material for: Multimodal fusion analysis of structural connectivity and gray matter morphology in migraine
Source: Hum Brain Mapp. 2020 Dec 8;42(4):908–21. doi: 10.1002/hbm.25267 (PMC7856653; doi:10.1002/hbm.25267)
Supplement: Supplementary file 2 — Appendix S2. Supporting Information. [file HBM-42-908-s002.pdf]

Supplementary File 2

In this file, the diverse mCCA-jICA steps are explained in more detail.

A. Input Data

The morphometric data, i.e., cortical curvature, cortical thickness, surface area and gray matter volume were considered as the first modality. From the Freesurfer pipeline, explained in detail in (Planchuelo-Gómez, García-Azorín, Guerrero, Rodríguez, et al., 2020), an 84-length vector for the gray matter volume, and a 68-length for the other features, were obtained for each subject. Each value from the vector corresponded to a specific region from the Desikan-Killiany atlas (Desikan et al., 2006). For each feature, the vectors from each group (HC, EM and CM) were arranged together and sorted following the group order. The input matrix for the next step, X_1, contained the values of the features in each subject, corresponding the first 50 rows to the HC values, the next 54 rows to EM, and the last 56 to CM. Each column from X_1 represented a specific region from the atlas.

The structural connectivity data, i.e., the number of streamlines between the reconstructed trajectories using tractography, were considered as the second modality. The full procedure to obtain a structural connectivity square matrix with the 84 regions from the Desikan-Killiany atlas is explained in (Planchuelo-Gómez, García-Azorín, Guerrero, Aja-Fernández, et al., 2020). We decided not to consider possible spurious connections. To that end, we discarded the connections with less than 1000 streamlines (the total number of streamlines per tractography was 10 million) in the three groups (group mean). Six hundred and twenty (620) connections survived the previous exclusion criterion. Furthermore, considering that cortical curvature, thickness and surface area only have values for cortical regions, connections where only subcortical

regions were involved were also discarded for the analysis were X_1 represented the data from one of the previous features. Therefore, for each subject, a 620-length vector or a 570-length vector were used depending on the gray matter morphometric parameter. The vectors from the subjects of each group were organized as explained in the previous paragraph to obtain X_2. Each column from X_2 represented a connection between two regions expressed as the number of streamlines. A graphical representation is shown:

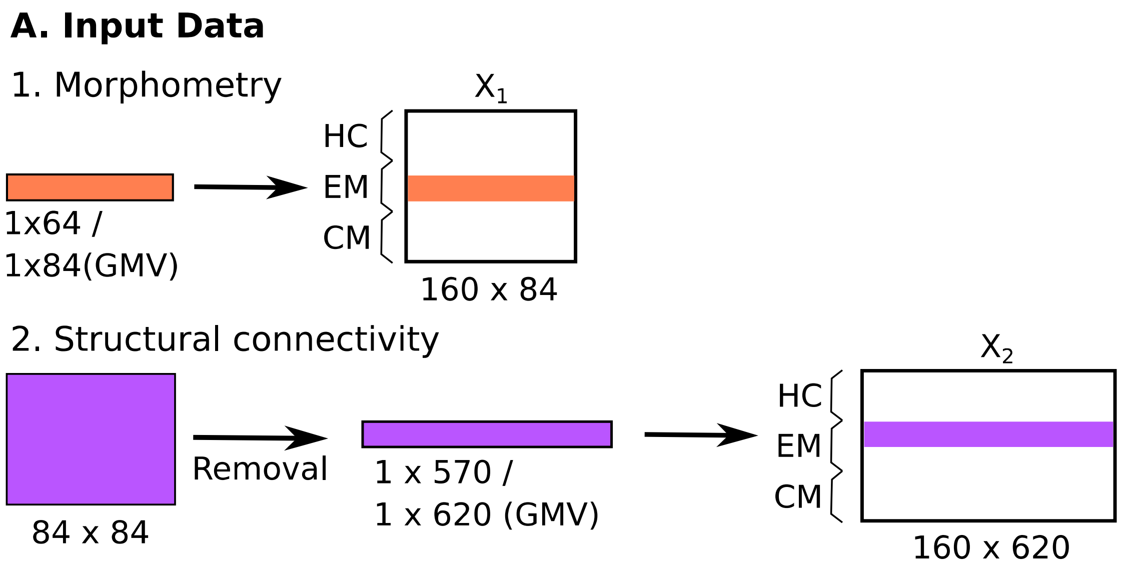

GMV = Gray Matter Volume. GMV is the gray matter feature taken as example. The number under each matrix represent its dimensions (rows *X* columns).

Overview of mCCA-jICA

Before explaining the full method, it is important to explain the main assumption from the whole process. As explained in (Kim et al., 2015; Sui et al., 2011), the multimodal dataset X_k is a linear mixture of sources S_k and the nonsingular matrix or mixing profile A_k ($k = \{1,2\}$):

$$X_k = A_k S_k \quad (1)$$

where A_k has dimension of subjects by number of components (N), and S_k has dimension of number of components by number of regions (k = 1) or connections (k = 2). A_k represents the contribution of each source to the corresponding feature set and its two columns are assumed to be highly correlated. The fusion of mCCA and jICA allows to overcome restrictions from both methods. On the one hand, with respect to mCCA, complete source separation is not normally reached for a small number of datasets (Sui et al., 2011), as in this case. On the other hand, the ICA model only allows one mixing profile instead of one mixing profile per modality. Thus, mCCA allows to have correlated components between diverse modalities and its results are the initialization of jICA, which allows to obtain independent components that alleviate the limitations of mCCA to reach a better source separation. The concept of the mixing profile is explained with more detail in step D.

B. mCCA

The objective of this step was to maximize the correlation between the analyzed modalities.

Following the methods described by (Sui et al., 2011), Singular Value Decomposition (SVD) was performed on X_k to reduce the dimension and discard noise or redundancy values. For the morphometric parameters, 99.7% of non-zero eigenvalues were retained for the gray matter volume (98.9% for the structural connectivity), 99.6% for the cortical curvature, 99.8% for the cortical thickness and 99.5% for the surface area (98.8% for the structural connectivity when combined with the last three parameters).

After SVD, the linear mixture model of mCCA was applied to compute the associated components:

$$X_k = D_k C_k \tag{2}$$

where D_k are the canonical variants and C_k the associated components (k = {1,2}). The canonical variants represent the contribution of the associated components to the individual features (Kim et al., 2015). The variants from each modality represent the mixing profile and have maximum correlation. The number of columns of D_k (and the number of rows of C_k) is equal to the minimum number of independent components between the modalities obtained with the Horn's test (Horn, 1965), explained in the main manuscript. The associated components represent the sources from each modality, but not a set of complete independent components or total source separation. A graphical representation is shown:

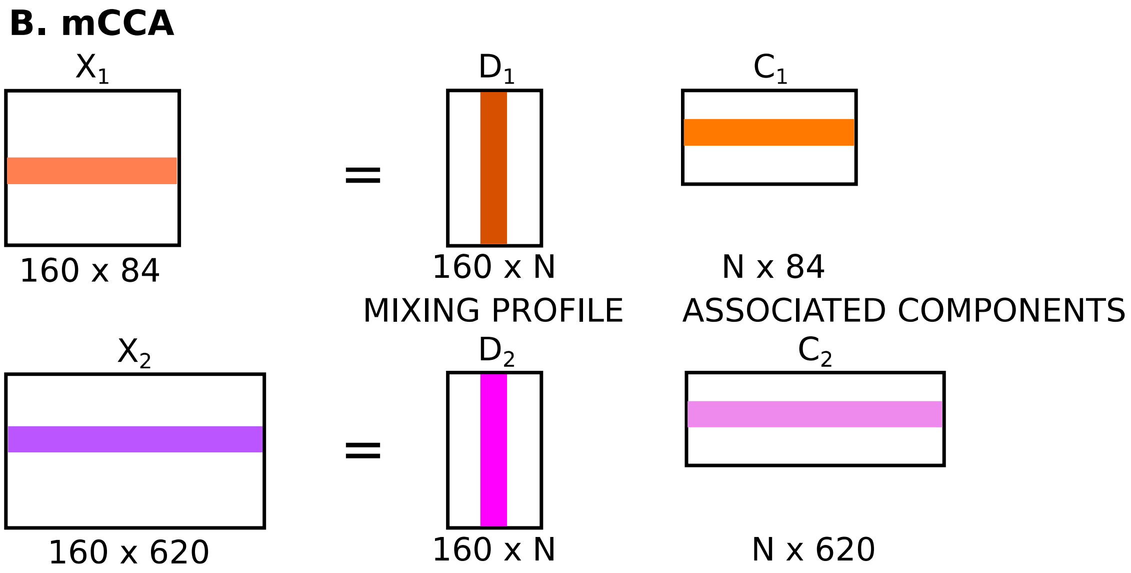

N = number of joint independent components. The mCCA step models the input data ($X_{1,2}$) from each modality as the product of the mixing profile ($D_{1,2}$) and the associated component ($C_{1,2}$).

C. jICA

The objective of this step was to maximize the independence of the associated components from mCCA and obtain independent sources for each modality.

In this step, the associated components from mCCA were concatenated and introduced as the input for jICA. The model of jICA was applied to calculate the independent sources or joint independent components in the following way:

$$C = W^{-1} S \qquad (3)$$

where C is the concatenation of C_1 and C_2, $[C_1, C_2]$, W^{-1} the pseudoinverse of the demixing matrix and S the concatenation of S_1 and S_2, $[S_1, S_2]$, the independent sources for each modality. W^{-1} represents the contribution of each independent source to its corresponding associated component. S has the same dimensions with respect to C, and W^{-1} is a square matrix with number of rows and columns equal to the number of rows of C and S, the number of independent components. A graphical representation is shown:

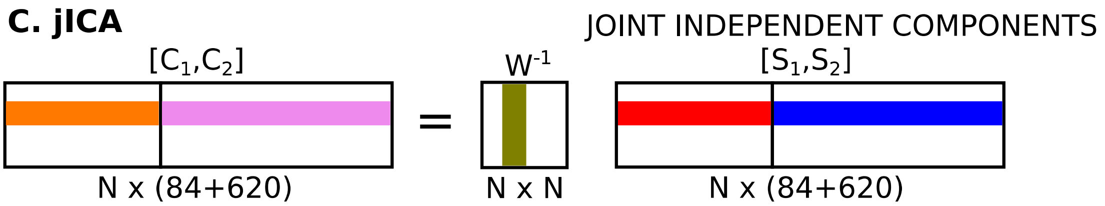

The jICA step models the concatenation of both associated components as the product of the demixing matrix (W^{-1}) and the joint independent components ($S_{1,2}$).

D. Mixing coefficients per modality

The objective of this step was to obtain a mixing profile per modality for all the subjects. The mixing matrix/profile per modality represents the contribution of the sources to the individual features of each modality. Considering similar sources between the groups of study (controls and patients with episodic and chronic migraine in our case), i.e., regions or connections which are highly represented in a source, the mixing matrix shows which subjects contribute more to the expression of the source. Grouping the mixing coefficients according to the groups of study, and assuming positive values of the Z-scores from the sources and the mixing coefficients, higher values of the mixing coefficients are directly related to higher values of the assessed feature. Therefore, for similar sources, the statistical comparison of the mixing coefficients is similar to the comparison of the features between the groups of interest in the regions or connections represented by the source. The interpretation of the results of the analysis is explained at the end of this file (before the references).

We show together equations (2) and (3), considering the results per modality in equation (3):

$$\begin{Bmatrix} X_k = D_k C_k \\ C_k = W^{-1} S_k \end{Bmatrix} \rightarrow X_k = D_k W^{-1} S_k. \tag{4}$$

To compute the mixing matrix per modality, we show together equation (4) and the equation from the whole mCCA-jICA method, equation (1):

$$\begin{Bmatrix} X_k = D_k W^{-1} S_k \\ X_k = A_k S_k \end{Bmatrix} \rightarrow A_k = D_k W^{-1} \tag{5}$$

where A_k is the mixing matrix per modality, i.e., the matrix with the mixing coefficients. The number of rows of A_k is equal to the number of subjects, and the number of columns to the number of independent components (same dimensions with respect to D_k). A graphical representation is shown:

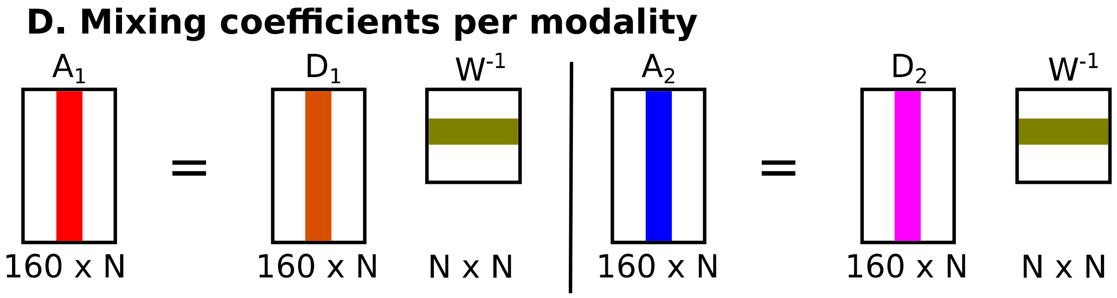

The mixing coefficients for each modality ($A_{1,2}$) are obtained by the product of the mixing profile and the demixing matrix.

The graphical representations from each step are displayed together:

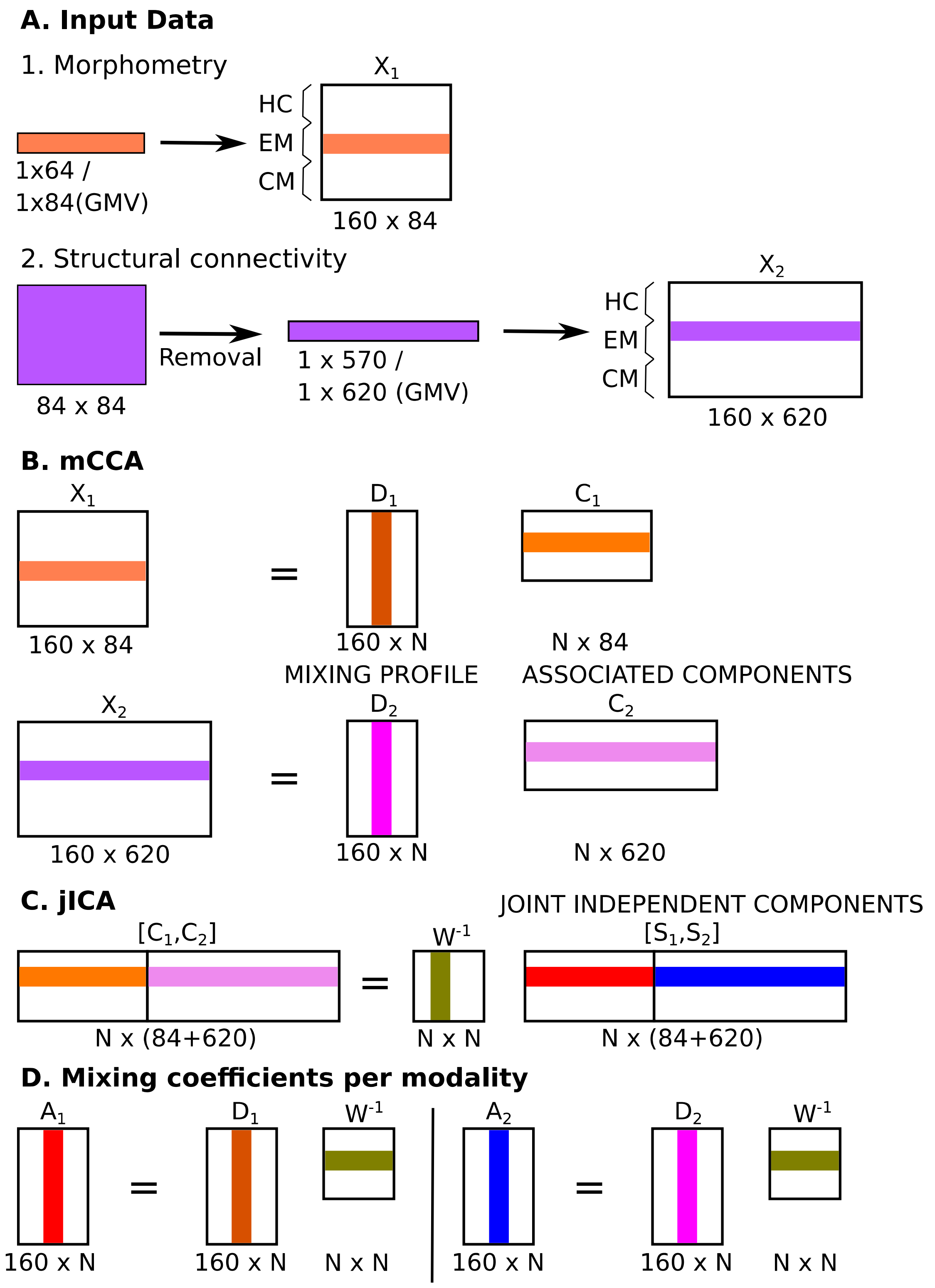

The mixing coefficients are compared between the three groups (HC, EM and CM) to identify differences in a morphometric parameter and/or structural connectivity. The Z-

scores from sources $S_{1,2}$ represent the weight of each region or connection to the joint independent component. Red and blue are used to indicate the values which are analyzed (statistics or outliers and Z-scores).

Analysis of mCCA-jICA

The results of this method were analyzed using the independent sources (S_k) and the mixing coefficients (A_k).

Each column of S_1 represented the value of a morphometric feature for a specific region, and each column of S_2 represented the value of the number of streamlines for a specific connection between two gray matter regions. The values of S_k were expressed as Z-scores. To collect the independent sources from each assessed group, we show equation (1) using the pseudoinverse matrix of the mixing matrix:

$$S_{Group,k} = A_{Group,k}^{-1} X_{Group,k} \quad (6)$$

where *Group* is equal to HC (first 50 rows of X_k and first 50 columns of A_k^{-1}), EM (next 54 positions) or CM (last 56 positions). The independent sources from each group were compared to analyze whether the same regions and connections were expressed in all the groups. If different regions or connections were expressed from one group to another, or if they were equal but expressed with opposite signs, i.e., negative Z-scores in one group and positive Z-scores in the other group, differences between groups would be found. If the regions or connections were equal, then the differences between groups would be analyzed using the mixing coefficients.

For the analysis of the mixing coefficients, each column of A_k (an independent component) was taken and the values were compared using statistical tests. Each

independent component contained the values of the three groups, corresponding the first 50 rows to HC, the next 54 to EM, and the last 56 to CM.

As explained in (Lottman et al., 2018) and the main manuscript, if the Z-scores from a source and the mixing coefficients were positive and significantly higher in one group with respect to another, the corresponding feature would be more expressed (higher values) in the group with higher coefficient values in the regions or connections represented by the source. If the Z-scores were negative and the mixing coefficients were positive and significantly higher in one group with respect to another, the feature would be less expressed (lower values) in the group with higher coefficient values in the regions/connections represented by the source. The opposite interpretation would be followed for negative mixing coefficients. Moreover, when significant differences were found in a specific component for both modalities, this component would be called "joint component". A modal-specific component would show significant differences in only one of the modalities.

REFERENCES

Desikan, R. S., Ségonne, F., Fischl, B., Quinn, B. T., Dickerson, B. C., Blacker, D., Buckner, R. L., Dale, A. M., Maguire, R. P., Hyman, B. T., Albert, M. S., & Killiany, R. J. (2006). An automated labeling system for subdividing the human cerebral cortex on MRI scans into gyral based regions of interest. *NeuroImage*, *31*(3), 968–980. https://doi.org/10.1016/j.neuroimage.2006.01.021

Horn, J. L. (1965). A rationale and test for the number of factors in factor analysis. *Psychometrika*, *30*(2), 179–185. https://doi.org/10.1007/BF02289447

Kim, S. G., Jung, W. H., Kim, S. N., Jang, J. H., & Kwon, J. S. (2015). Alterations of Gray and White Matter Networks in Patients with Obsessive-Compulsive Disorder: A Multimodal Fusion Analysis of Structural MRI and DTI Using mCCA+jICA. *PLoS ONE*, *10*(6), e0127118. https://doi.org/10.1371/journal.pone.0127118

Lottman, K. K., White, D. M., Kraguljac, N. V, Reid, M. A., Calhoun, V. D., Catao, F., & Lahti, A. C. (2018). Four-way multimodal fusion of 7 T imaging data using an mCCA+jICA model in first-episode schizophrenia. *Hum Brain Mapp*, *39*(4), 1475–1488. https://doi.org/10.1002/hbm.23906

Planchuelo-Gómez, Á., García-Azorín, D., Guerrero, Á. L., Aja-Fernández, S., Rodríguez, M., & de Luis-García, R. (2020). Structural connectivity alterations in chronic and episodic migraine: A diffusion magnetic resonance imaging connectomics study. *Cephalalgia*, *40*(4), 367–383. https://doi.org/10.1177/0333102419885392

Planchuelo-Gómez, Á., García-Azorín, D., Guerrero, Á. L., Rodríguez, M., Aja-Fernández, S., & de Luis-García, R. (2020). Grey matter structural alterations in chronic and episodic migraine: a morphometric magnetic resonance imaging study.

Pain Medicine. https://doi.org/10.1093/pm/pnaa271

Sui, J., Pearlson, G., Adali, T., Kiehl, K. A., Caprihan, A., Liu, J., Yamamoto, J., & Calhoun, V. D. (2011). Discriminating Schizophrenia and Bipolar Disorder by Fusing FMRI and DTI in A Multimodal CCA+ Joint ICA Model. *NeuroImage*, *57*(3), 839–855. https://doi.org/10.1016/j.neuroimage.2011.05.055
