# Supplementary material for: Multimodal fusion analysis of structural connectivity and gray matter morphology in migraine
Source: Hum Brain Mapp. 2020 Dec 8;42(4):908–21. doi: 10.1002/hbm.25267 (PMC7856653; doi:10.1002/hbm.25267)
Supplement: Supplementary file 3 — Appendix S3. Supporting Information. [file HBM-42-908-s003.pdf]

Supplementary File 3

Supplementary Table 1. Networks with positive jICA outlier values (Z > 0) in controls from the fusion of cortical curvature and structural connectivity**.**

Hemisphere	Network regions
Left	Precentral – Superior parietal – Postcentral – Supramarginal
Right	Supramarginal – Inferior parietal – Precuneus – Superior parietal – Postcentral – Paracentral – Precentral
Left	Pericalcarine – Lateral occipital – Fusiform – Superior temporal
Right	Caudal middle frontal – Rostral middle frontal – Superior frontal
Right	Transverse temporal – Postcentral – Hippocampus – Fusiform – Superior temporal – Transverse temporal
Right	Pars orbitalis – Rostral middle frontal – Pars triangularis
Right	Inferior temporal – Fusiform – Lingual

No inter hemispheric connections were detected as outliers.

Supplementary Table 2. Networks with positive jICA outlier values (Z > 0) in EM patients from the fusion of cortical curvature and structural connectivity.

Hemisphere	Network regions
Left	Precuneus – Superior parietal – Postcentral – Precentral – Supramarginal
Right	Caudal middle frontal – Precentral – Paracentral – Superior parietal – Inferior parietal – Supramarginal – Postcentral
Left	Lateral occipital – Fusiform – Inferior temporal
Left/Right	Medial orbito frontal – Lateral orbito frontal – Caudate
Left/Right	Medial orbito frontal – Lateral orbito frontal – Putamen – Insula

No inter hemispheric connections were detected as outliers. The three underlined regions mean that all possible pairs of regions are connected between them.

Supplementary Table 3. Networks with negative jICA outlier values (Z < 0) in CM patients from the fusion of cortical curvature and structural connectivity.

Hemisphere	Network regions
Left	Cuneus – Lateral occipital – Lingual
Right	Cuneus – Lateral occipital – Precuneus
Left	Banks of the superior temporal sulcus – Superior temporal – Middle temporal
Left	Medial orbito frontal – Lateral orbito frontal – Caudate

No inter hemispheric connections were detected as outliers.

Supplementary Table 4. Networks with positive jICA outlier values (Z > 0) in CM patients from the fusion of cortical curvature and structural connectivity.

Hemisphere	Network regions
Left	Parahippocampal – Hippocampus – Fusiform – Superior temporal – Transverse temporal
Right	Parahippocampal – Hippocampus – Insula – Putamen
Left	Insula – Putamen – Lateral orbito frontal – Rostral middle frontal – Superior frontal
Left	Medial orbito frontal – Putamen – Lateral orbito frontal – Rostral middle frontal – Superior frontal
Left	Rostral anterior cingulate – Putamen – Lateral orbito frontal – Rostral middle frontal – Superior frontal
Right	Caudal anterior cingulate – Superior frontal – Rostral middle frontal
Right	Inferior parietal – Superior parietal – Pericalcarine
Right	Inferior temporal – Superior temporal – Fusiform – Lateral occipital
Right	Inferior temporal – Superior temporal – Fusiform – Lingual
Right	Inferior temporal – Superior temporal – Transverse temporal

No inter hemispheric connections were detected as outliers.

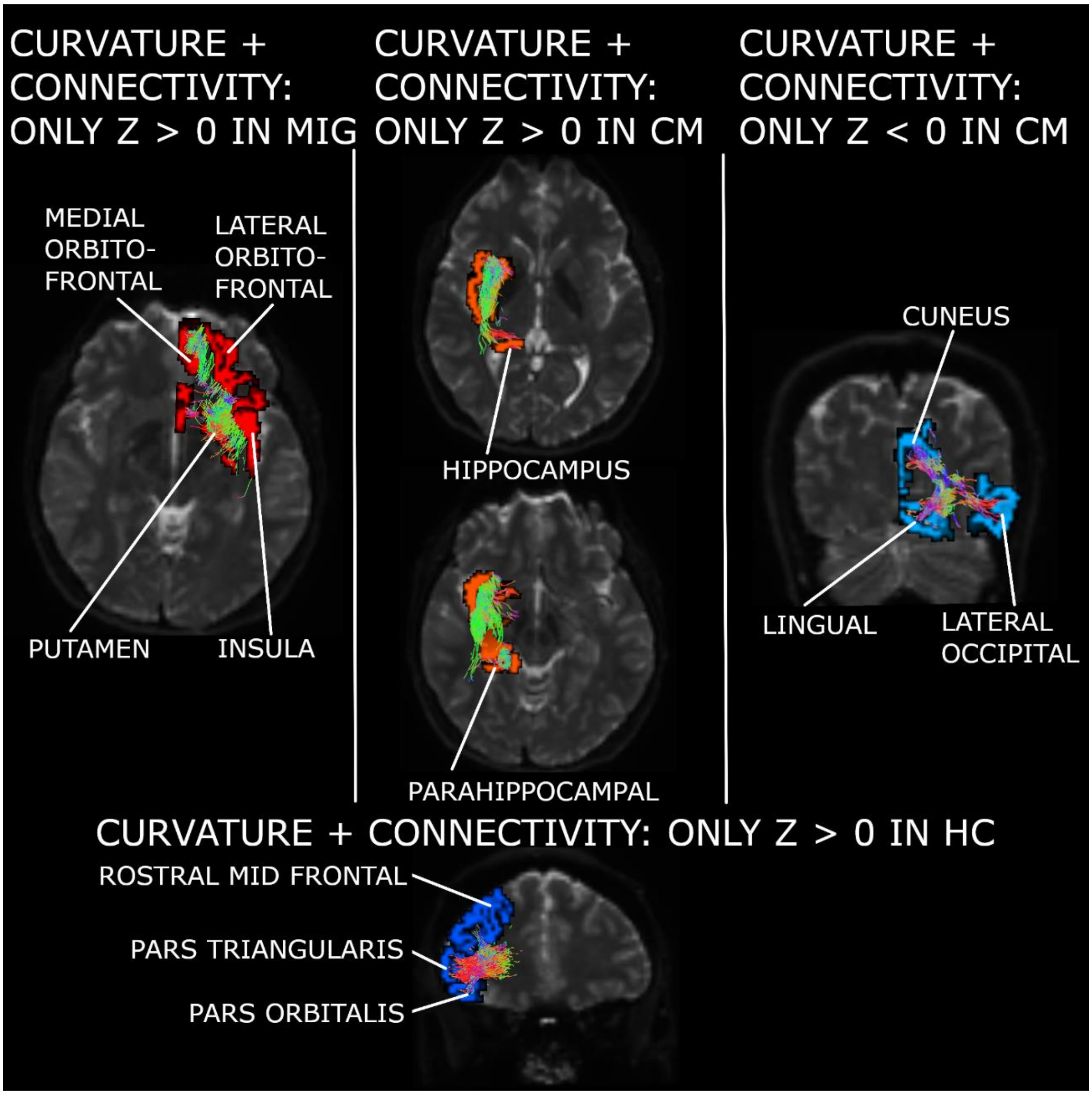

Supplementary Figure 1 Additional networks found for the first independent component (fusion of curvature and connectivity). Networks enhanced or worsened only in migraine, CM or HC are shown. MID = middle; MIG = migraine.

Supplementary Table 5. Networks with positive jICA outlier values (Z > 0) in controls from the fusion of cortical thickness and structural connectivity.

Hemisphere	Network regions
Left	Precuneus – Superior parietal – Supramarginal
Left	Inferior parietal – Superior parietal – Supramarginal
Right	Precuneus – Superior parietal – Inferior parietal – Lateral occipital – Fusiform – Inferior temporal
Right	Lingual – Lateral occipital – Fusiform – Inferior temporal
Left	Medial orbito frontal – Lateral orbito frontal – Rostral middle frontal – Pars orbitalis
Left	Medial orbito frontal – Lateral orbito frontal – Rostral middle frontal – Pars triangularis
Left	Middle temporal – Inferior temporal – Superior temporal
Left	Fusiform – Inferior temporal – Superior temporal
Right	Superior parietal – Cuneus – Pericalcarine

No inter hemispheric connections were detected as outliers. Three underlined regions mean that all possible pairs of regions are connected between them.

Supplementary Table 6. Networks with negative jICA outlier values (Z < 0) in EM patients from the fusion of cortical thickness and structural connectivity.

Hemisphere	Network regions
Right	Paracentral – Precentral – Superior parietal – Postcentral
Right	Paracentral – Precentral – Superior parietal – Pericalcarine

No inter hemispheric connections were detected as outliers.

Supplementary Table 7. Networks with positive jICA outlier values (Z > 0) in EM patients from the fusion of cortical thickness and structural connectivity.

Hemisphere	Network regions
Left	Precuneus – Superior parietal – Inferior parietal – Supramarginal
Left	Banks of the superior temporal sulcus – Middle temporal – Inferior temporal – Superior temporal – Fusiform
Right	Banks of the superior temporal sulcus – Middle temporal – Inferior temporal – Superior temporal – Transverse temporal
Right	Fusiform – Insula – Inferior temporal – Middle temporal
Left	Cuneus – Lateral occipital – Lingual
Left/Right	<u>Cuneus – Lateral occipital – Pericalcarine</u>
Right	Inferior Parietal – Pericalcarine – Lingual
Right	Medial orbito frontal – Lateral orbito frontal – Rostral middle frontal

No inter hemispheric connections were detected as outliers. Three underlined regions mean that all possible pairs of regions are connected between them.

Supplementary Table 8. Networks with negative jICA outlier values (Z < 0) in CM patients from the fusion of cortical thickness and structural connectivity.

Hemisphere	Network regions
Left	Caudal middle frontal – Precentral – Superior parietal
Left	Superior temporal – Isthmus cingulate – Thalamus
Left	Superior temporal – Isthmus cingulate – Hippocampus

No inter hemispheric connections were detected as outliers.

Supplementary Table 9. Networks with positive jICA outlier values (Z > 0) in CM patients from the fusion of cortical thickness and structural connectivity.

Hemisphere	Network regions
Left	Inferior parietal – Precuneus – Superior parietal – Cuneus
Left	Lateral occipital – Superior parietal - Supramarginal
Right	Precuneus – Superior parietal – Cuneus – Lateral occipital – Inferior parietal
Left	Insula – Fusiform – Inferior temporal – Middle temporal – Superior temporal – Banks of the superior temporal sulcus
Right	Superior temporal – Inferior temporal – Middle temporal
Right	Insula – Inferior temporal – Fusiform

No inter hemispheric connections were detected as outliers. Three underlined regions mean that all possible pairs of regions are connected between them.

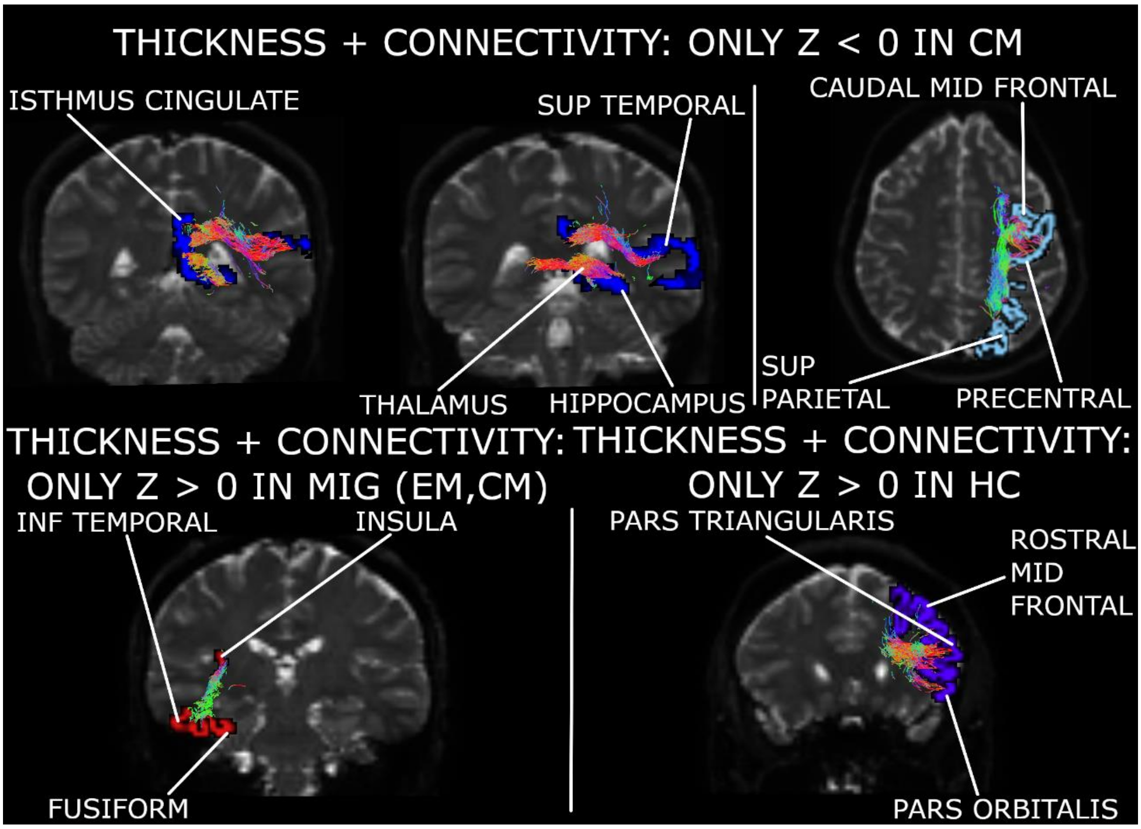

Supplementary Figure 2 Additional networks found for the fifth independent component (fusion of thickness and connectivity). Networks enhanced or worsened only in migraine, CM or HC are shown. INF = inferior; MID = middle; MIG = migraine (results from EM and CM); SUP = superior.

Supplementary Table 10. Networks with negative jICA outlier values (Z < 0) in controls from the fusion of gray matter volume and structural connectivity.

Hemisphere	Network regions
Left	Paracentral – Precentral – Caudate – Thalamus – Putamen – Hippocampus
Left	Putamen – Lateral orbito frontal – Caudate
Left	Cuneus – Superior parietal – Postcentral
Right	Postcentral – Paracentral – Precentral

No inter hemispheric connections were detected as outliers.

Supplementary Table 11. Networks with positive jICA outlier values (Z > 0) in controls from the fusion of gray matter volume and structural connectivity.

Hemisphere	Network regions
Left	Banks of the superior temporal sulcus – Middle temporal – Inferior temporal
Left	Medial orbito frontal – Lateral orbito frontal – Rostral middle frontal

No inter hemispheric connections were detected as outliers.

Supplementary Table 12. Networks with negative jICA outlier values (Z < 0) in EM patients from the fusion of gray matter volume and structural connectivity.

Hemisphere	Network regions
Left	Banks of the superior temporal sulcus – Middle temporal – Inferior temporal – Superior temporal – Fusiform
Right	Banks of the superior temporal sulcus – Middle temporal – Inferior temporal – Insula – Fusiform
Left	Lateral orbito frontal – Rostral middle frontal – Pars triangularis
Left	Pars orbitalis – Rostral middle frontal – Pars triangularis
Right	Lateral orbito frontal – Rostral middle frontal – Pars orbitalis
Left	Inferior parietal – Supramarginal – Postcentral
Right	Cuneus – Pericalcarine – Lateral occipital - Precuneus

No inter hemispheric connections were detected as outliers. The three underlined regions mean that all possible pairs of regions are connected between them.

Supplementary Table 13. Networks with positive jICA outlier values (Z > 0) in EM patients from the fusion of gray matter volume and structural connectivity.

Hemisphere	Network regions
Left	Thalamus – Caudate – Lateral orbito frontal – Putamen – Insula
Right	Precentral – Thalamus – Caudate – Lateral orbito frontal
Right	Precentral – Thalamus – Caudate – Rostral anterior cingulate
Right	Pericalcarine – Superior parietal – Postcentral
Right	Paracentral – Superior parietal – Postcentral

No inter hemispheric connections were detected as outliers. The three underlined regions mean that all possible pairs of regions are connected between them.

Supplementary Table 14. Networks with negative jICA outlier values (Z < 0) in CM patients from the fusion of gray matter volume and structural connectivity.

Hemisphere	Network regions
Left	Cuneus – Lateral occipital – Inferior parietal
Left	Cuneus – Lateral occipital – Lingual
Right	Cuneus – Pericalcarine – Lateral occipital – Precuneus
Left	Banks of the superior temporal sulcus – Superior temporal – Inferior temporal – Middle temporal
Right	Superior temporal – Inferior temporal – Middle temporal
Right	Lateral orbito frontal – Rostral middle frontal – Pars orbitalis
Right	Caudal middle frontal – Superior frontal – Paracentral
Right	Pallidum – Insula – Fusiform

No inter hemispheric connections were detected as outliers. Three underlined regions mean that all possible pairs of regions are connected between them.

Table S15. Networks with positive jICA outlier values (Z > 0) in CM patients from the fusion of gray matter volume and structural connectivity.

Hemisphere	Network regions
Left	Thalamus – Caudate – Precentral
Right	Thalamus – Caudate – Putamen
Left	Lateral orbito frontal – Putamen – Medial orbito frontal
Right	Rostral anterior cingulate – Putamen – Insula

No inter hemispheric connections were detected as outliers.

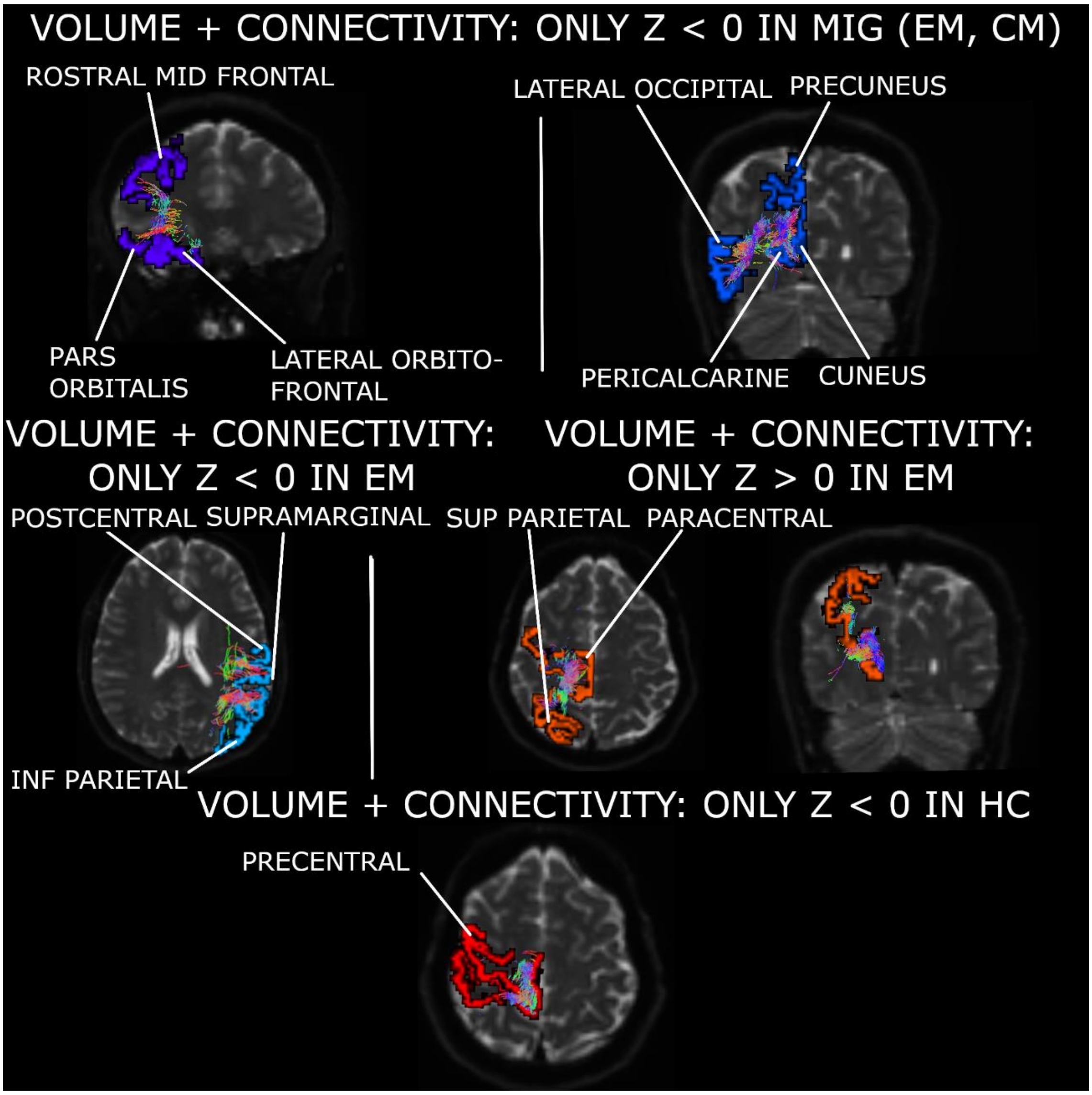

Supplementary Figure 3 Additional networks found for the third independent component (fusion of gray matter volume and connectivity), part 1. Networks enhanced or worsened only in migraine, EM or HC are shown. INF = inferior; MID = middle; MIG = migraine (results from EM and CM); SUP = superior.

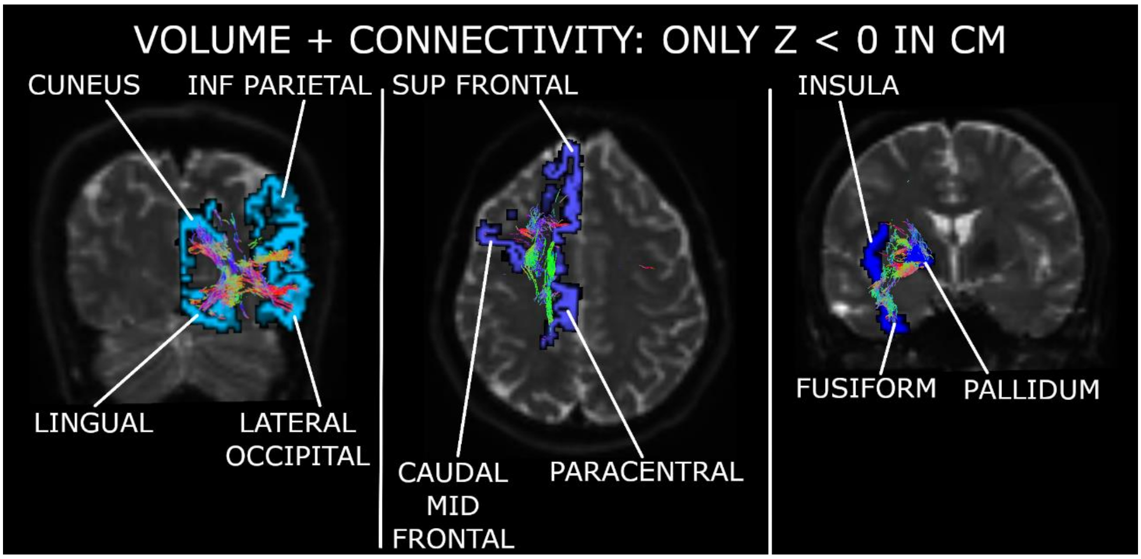

Supplementary Figure 4 Additional networks found for the third independent component (fusion of gray matter volume and connectivity), part 2. Networks worsened only in CM are shown. INF = inferior; MID = middle; SUP = superior.
